# Supplementary material for: Elimination of MYCN-Amplified Neuroblastoma Cells by Telomerase-Targeted Oncolytic Virus via MYCN Suppression
Source: Mol Ther Oncolytics. 2020 Jun 1;18:14–23. doi: 10.1016/j.omto.2020.05.015 (PMC7321810; doi:10.1016/j.omto.2020.05.015)
Supplement: Document S1. Figures S1–S8 [file mmc1.pdf]

OMTO, Volume *18*

Supplemental Information

Elimination of MYCN-Amplified Neuroblastoma Cells by Telomerase-Targeted Oncolytic Virus via MYCN Suppression

Terutaka Tanimoto, Hiroshi Tazawa, Takeshi Ieda, Hiroshi Nouso, Morimichi Tani, Takanori Oyama, Yasuo Urata, Shunsuke Kagawa, Takuo Noda, and Toshiyoshi Fujiwara

SUPPLEMENTARY MATERIAL

Elimination of MYCN-amplified neuroblastoma cells by telomerase-targeted oncolytic virus via MYCN suppression

Terutaka Tanimoto[1,2], Hiroshi Tazawa[1,3], Takeshi Ieda[1], Hiroshi Nouso[2], Morimichi Tani[2], Takanori Oyama[2], Yasuo Urata[5], Shunsuke Kagawa[1,3], Takuo Noda[2], and Toshiyoshi Fujiwara[1]

Departments of [1]Gastroenterological Surgery and [2]Pediatric Surgery, Okayama University Graduate School of Medicine, Dentistry and Pharmaceutical Sciences, Okayama 700-8558, Japan. [3]Center for Innovative Clinical Medicine and [4]Minimally Invasive Therapy Center, Okayama University Hospital, Okayama 700-8558, Japan.
[5]Oncolys BioPharma, Inc., Tokyo, 106-0032, Japan.

Figure S1
In vitro cytopathic effect of OBP-301 and OBP-702
against NB-1 and LA-N-5 cells.

Figure S2
In vitro cytopathic effect of OBP-301 and OBP-702
against IMR-32 and LA-N-5 cells.

Figure S3
Expression of hTERT mRNA
in human NB cells with or without MYCN amplification.

Figure S4
In vitro cytopathic effect of OBP-301 and OBP-702
against non-MYCN-amplified SK-N-SH cells.

Figure S5
Inverse correlation between E2F1 protein and MYCN mRNA expression.

Figure S6
MYCN is not downregulated in IMR-32 and CHP-134 cells
infected with dl312.

Figure S7
No antitumor effect of OBP-301 and OBP-702
in a subcutaneous CHP-134 xenograft tumor model.

Figure S8
Histologic analysis of CHP-134 tumors.

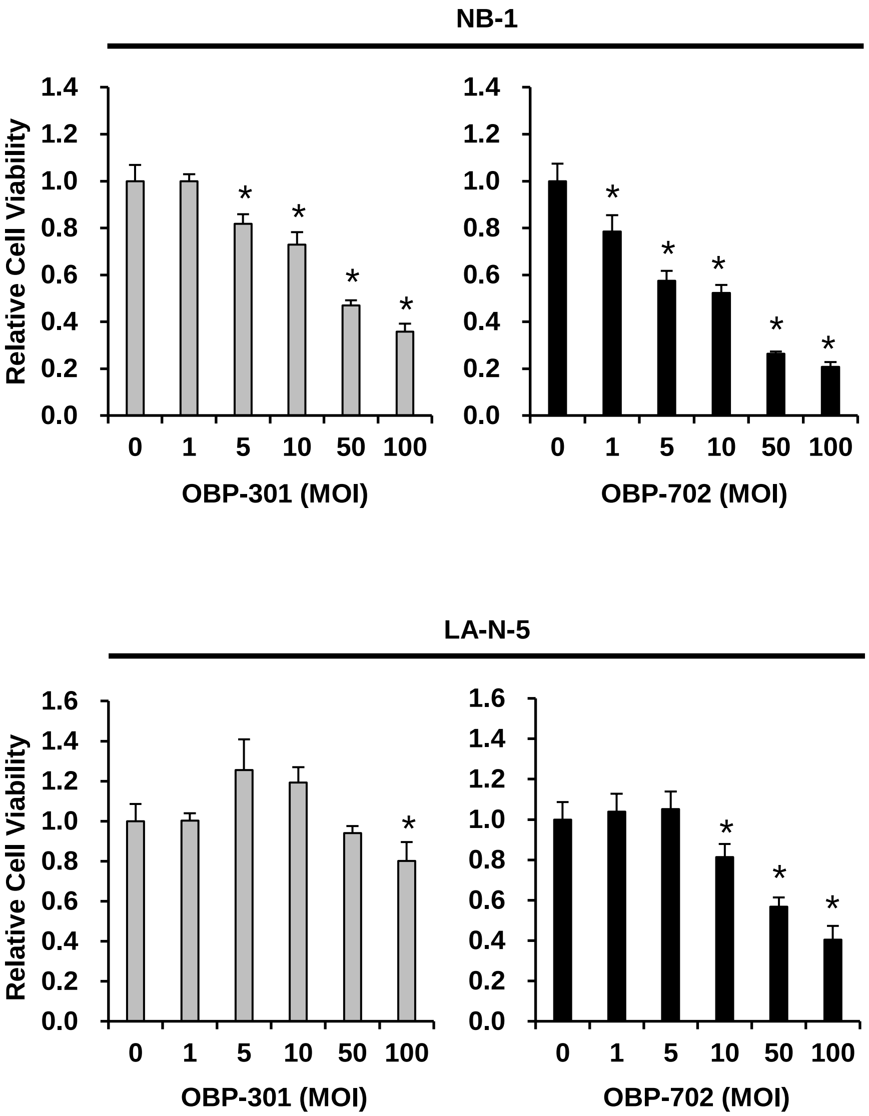

Figure S1

In vitro cytopathic effect of OBP-301 and OBP-702 against NB-1 and LA-N-5 cells.

NB-1 and LA-N-5 cells were infected with OBP-301 or OBP-702 at the indicated MOI, and cell viability was evaluated on day 3 after infection using an XTT assay . Cell viability was calculated relative to that of mock-infected cells, which was set at 1.0. Cell viability data are expressed as mean ± SD (n = 5). *: $P < 0.05$ (vs 0 MOI).

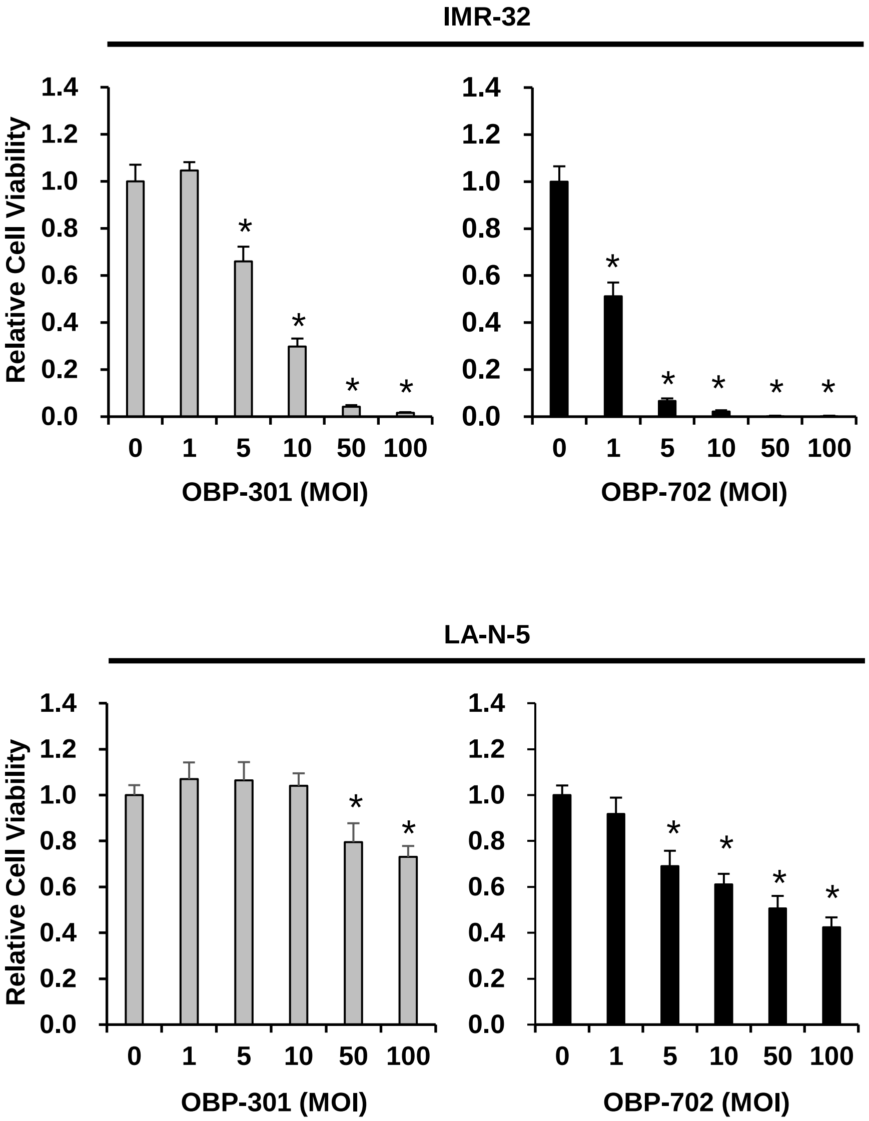

Figure S2

***In vitro* cytopathic effect of OBP-301 and OBP-702 against IMR-32 and LA-N-5 cells.**

IMR-32 and LA-N-5 cells were infected with OBP-301 or OBP-702 at the indicated MOI, and cell viability was evaluated on day 5 after infection using an XTT assay . Cell viability was calculated relative to that of mock-infected cells, which was set at 1.0. Cell viability data are expressed as mean ± SD (n = 5). *: $P < 0.05$ (vs 0 MOI).

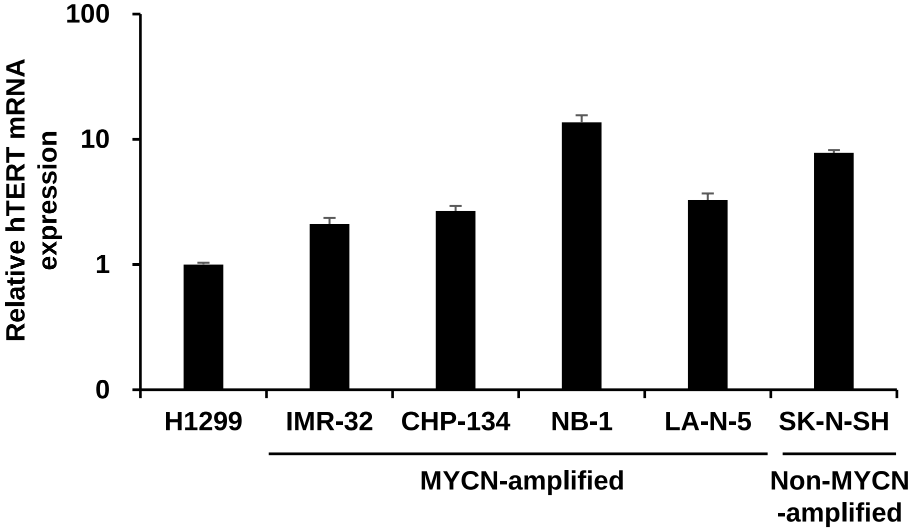

Figure S3

Expression of hTERT mRNA in human NB cells with or without MYCN amplification.

Expression of hTERT mRNA was analyzed using qRT-PCR. The expression level of hTERT mRNA was calculated relative to that of hTERT mRNA in H1299 cells, which was set at 1. Data are expressed as mean ± SD (n = 3).

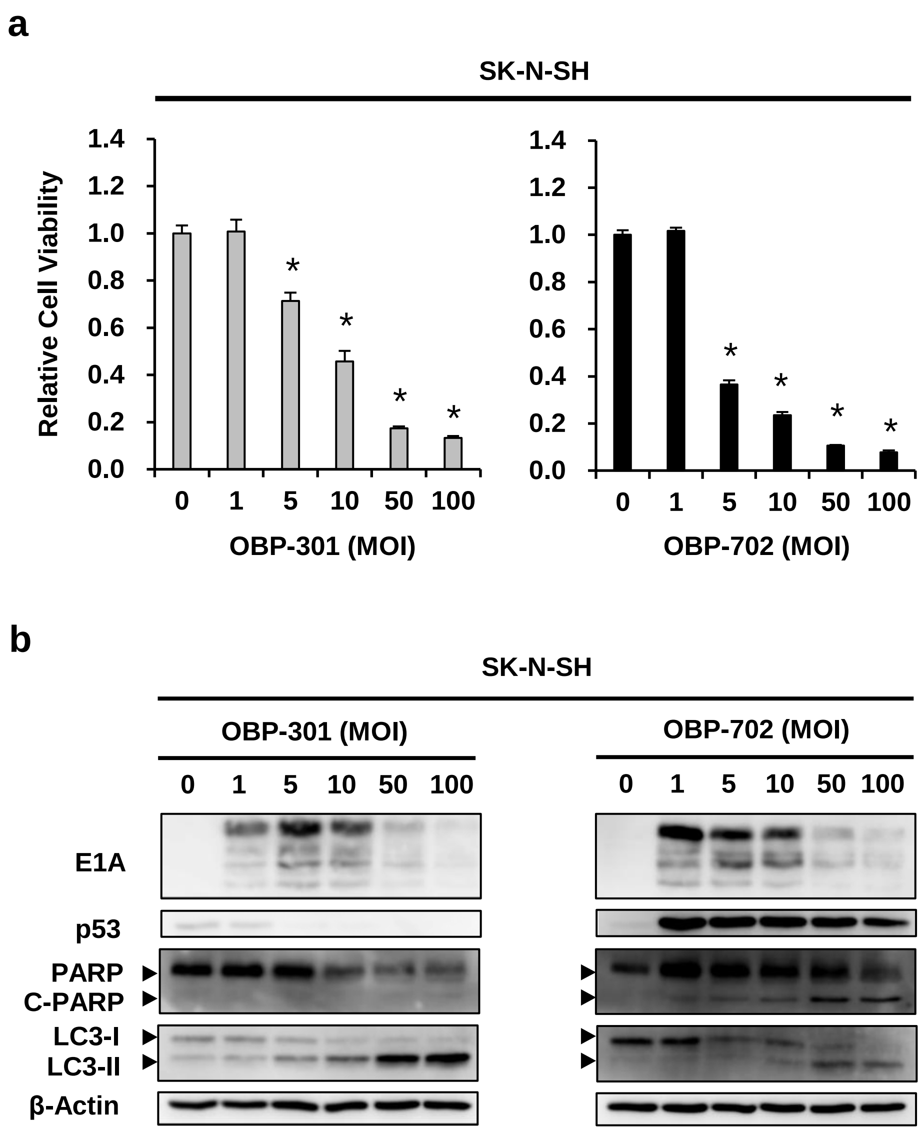

Figure S4

***In vitro* cytopathic effect of OBP-301 and OBP-702 in non-MYCN-amplified SK-N-SH cells.**

(a) SK-N-SH cells were infected with OBP-301 or OBP-702 at the indicated MOI, and cell viability was evaluated on day 3 after infection using an XTT assay. Cell viability was calculated relative to that of mock-infected cells, which was set at 1.0. Cell viability data are expressed as mean ± SD (n = 5). *: $P < 0.05$ (vs 0 MOI). (b) Expression of viral E1A, p53, PARP, cleaved PARP (C-PARP), and LC3 protein in SK-N-SH cells infected with OBP-301 or OBP-702 at the indicated MOI for 72 h. β-Actin was assayed as a loading control.

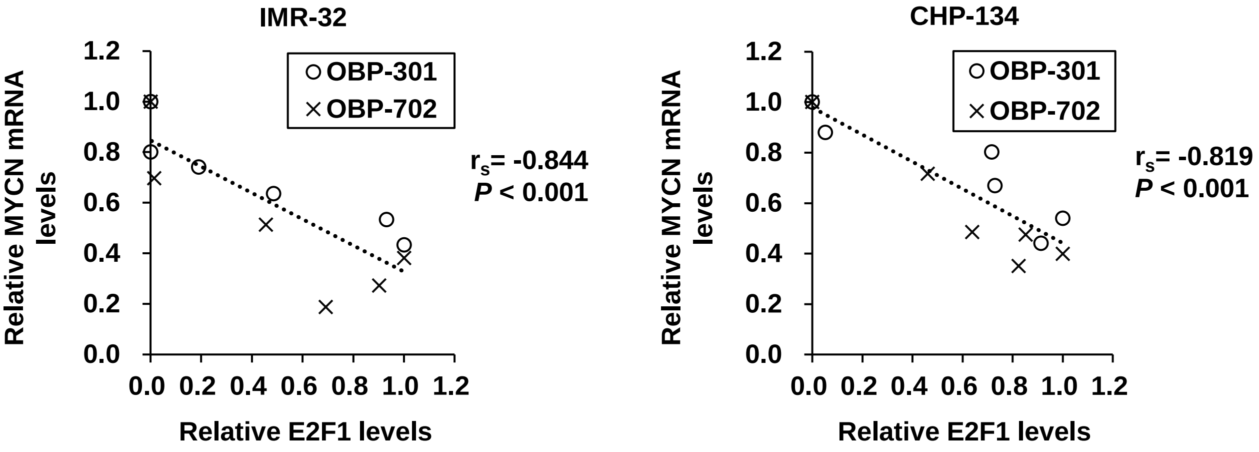

Figure S5

Inverse correlation between E2F1 protein and MYCN mRNA expression.

There was significant correlation between the expression levels of E2F1 protein and MYCN mRNA in IMR-32 and CHP-134 cells. The expression level of E2F1 protein was calculated relative to that of the most E2F1-active cells, which was set at 1.0. The expression level of MYCN mRNA was calculated relative to that of mock-infected cells, which was set at 1.0.

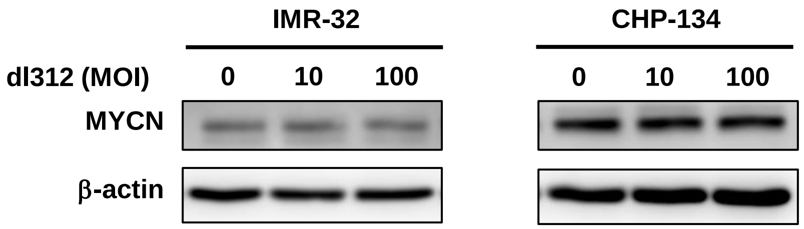

Figure S6

MYCN is not downregulated in IMR-32 and CHP-134 cells infected with dl312.

Expression of MYCN protein in IMR-32 and CHP-134 cells infected with dl312 at the indicated MOI for 72 h. β-Actin was assayed as a loading control.

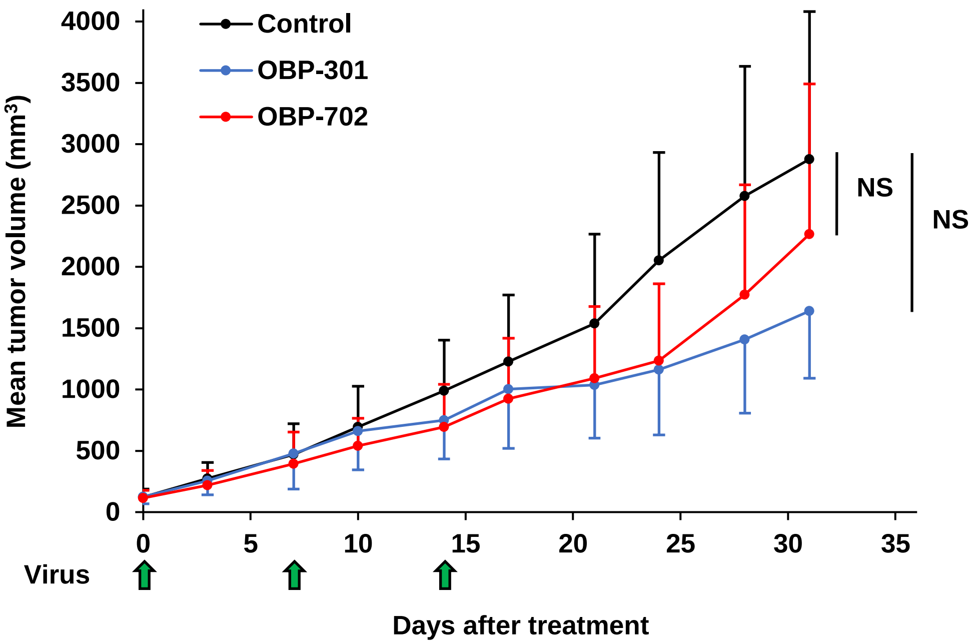

Figure S7

No antitumor effect of OBP-301 and OBP-702 in a subcutaneous CHP-134 xenograft tumor model.

CHP-134 cells (1×10^7 cells) were inoculated into the flank of 6-week-old female BALB/c *nu/nu* mice. OBP-301 (10^8 PFU), OBP-702 (10^8 PFU), or PBS (Mock) was intratumorally injected one time a week for 3 cycles. Tumor growth is expressed as mean tumor volume $\pm$ SD (n = 8-10 in each group; NS, not significant).

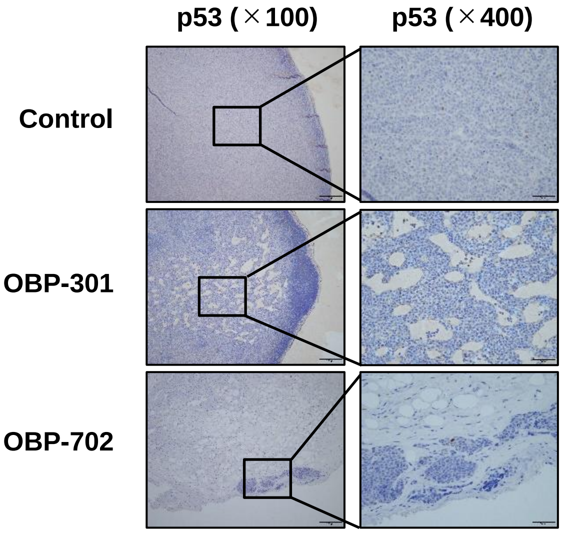

Figure S8

Histologic analysis of CHP-134 tumors.

Paraffin-embedded sections of CHP-134 tumors were stained with anti-p53 antibody. Left-side images in each figure are low magnification, and right-side images are high magnification of the area outlined by a black square. Left scale bars, 200 µm. Right scale bars, 100 µm.
