# Supplementary material for: Elimination of MYCN-Amplified Neuroblastoma Cells by Telomerase-Targeted Oncolytic Virus via MYCN Suppression
Source: Mol Ther Oncolytics. 2020 Jun 1;18:14–23. doi: 10.1016/j.omto.2020.05.015 (PMC7321810; doi:10.1016/j.omto.2020.05.015)
Supplement: Document S2. Article plus Supplemental Information [file mmc2.pdf]

Molecular Therapy
Oncolytics
Original Article

Elimination of MYCN-Amplified Neuroblastoma Cells by Telomerase-Targeted Oncolytic Virus via MYCN Suppression

Terutaka Tanimoto,[1,2] Hiroshi Tazawa,[1,3] Takeshi Ieda,[1] Hiroshi Nouso,[2] Morimichi Tani,[2] Takanori Oyama,[2] Yasuo Urata,[5] Shunsuke Kagawa,[1,4] Takuo Noda,[2] and Toshiyoshi Fujiwara[1]

[1]Department of Gastroenterological Surgery, Okayama University Graduate School of Medicine, Dentistry and Pharmaceutical Sciences, Okayama 700-8558, Japan; [2]Department of Pediatric Surgery, Okayama University Graduate School of Medicine, Dentistry and Pharmaceutical Sciences, Okayama 700-8558, Japan; [3]Center for Innovative Clinical Medicine, Okayama University Hospital, Okayama 700-8558, Japan; [4]Minimally Invasive Therapy Center, Okayama University Hospital, Okayama 700-8558, Japan; [5]Oncolys BioPharma, Inc., Tokyo 106-0032, Japan

Neuroblastoma (NB) is a primary malignant tumor of the peripheral sympathetic nervous system. High-risk NB is characterized by *MYCN* amplification and human telomerase reverse transcriptase (*hTERT*) rearrangement, contributing to hTERT activation and a poor outcome. For targeting hTERT-activated tumors, we developed two oncolytic adenoviruses, OBP-301 and tumor suppressor p53-armed OBP-702, in which the hTERT promoter drives expression of the viral *E1* gene for tumor-specific virus replication. In this study, we demonstrate the therapeutic potential of the hTERT-driven oncolytic adenoviruses OBP-301 and OBP-702 using four human MYCN-amplified NB cell lines (IMR-32, CHP-134, NB-1, LA-N-5) exhibiting high hTERT expression. OBP-301 and OBP-702 exhibited a strong antitumor effect in association with autophagy in NB cells. Virus-mediated activation of E2F1 protein suppressed MYCN expression. OBP-301 and OBP-702 significantly suppressed the growth of subcutaneous CHP-134 tumors. Thus, these hTERT-driven oncolytic adenoviruses are promising antitumor agents for eliminating MYCN-amplified NB cells via E2F1-mediated suppression of MYCN protein.

INTRODUCTION

Neuroblastoma (NB) is a malignancy of the peripheral sympathetic nervous system.[1] NB is the most common extracranial solid tumor, accounting for 15% of all childhood cancer deaths.[2] The biological behavior of NB differs considerably between low-risk and high-risk NB tumors. Low-risk NB tumors spontaneously disappear or differentiate into benign tumors, whereas high-risk NB tumors rapidly progress and exhibit an invasive and metastatic phenotype.[3] Amplification of the *MYCN* oncogene is one of the most critical prognostic factors in high-risk NB tumors.[4] Because MYCN is strongly associated with progressive disease and unfavorable prognosis in high-risk NB,[4] MYCN is thought to play a central role in maintaining the malignant potential of high-risk NB tumors, suggesting that MYCN is an attractive therapeutic target for the treatment of this disease.[5] However, it has been extremely difficult to develop a specific inhibitor that directly targets MYCN protein in high-risk NB.[6] In contrast, the results of recent comprehensive genomic analyses suggested that human telomerase reverse transcriptase (*hTERT*) rearrangement is a novel prognostic factor in high-risk NB tumors.[7,8] Interestingly, high-risk NB tumors exhibiting MYCN amplification and hTERT rearrangement have similar characteristics with transcriptional activation of hTERT expression. Therefore, high hTERT expression may be a novel therapeutic target for the treatment of high-risk NB involving MYCN amplification and/or hTERT rearrangement.

Oncolytic virotherapy has recently emerged as a novel antitumor modality for inducing specific death of tumor cells without affecting normal cells.[9] To target hTERT-activated malignant tumor cells, we developed two types of hTERT-driven oncolytic adenoviruses, OBP-301 (Telomelysin)[10] and tumor suppressor p53-expressing OBP-702,[11] in which the hTERT promoter drives the E1 gene for tumor-specific virus replication. OBP-301 and OBP-702 demonstrated therapeutic potential for inducing lytic death in association with autophagy and apoptosis in a variety of human cancer cells expressing hTERT.[11–14] A phase I clinical trial conducted in patients with advanced solid tumors in the United States showed that OBP-301 was well tolerated by patients.[15] In addition, a preclinical study demonstrated that OBP-301 sensitizes tumor cells to radiotherapy,[16] and a phase I/II study of OBP-301 in combination with radiotherapy is currently ongoing in patients with esophageal cancer. Because high-risk NB tumors exhibit high hTERT expression, hTERT-targeted oncolytic virotherapy may be an ideal antitumor strategy for the treatment of this disease.

In the present study, we investigated the therapeutic potential of the hTERT-driven oncolytic adenoviruses OBP-301 and OBP-702 against MYCN-amplified NB cells. To elucidate the molecular mechanism

Received 1 May 2020; accepted 27 May 2020;
https://doi.org/10.1016/j.omto.2020.05.015.

Correspondence: Hiroshi Tazawa, Center for Innovative Clinical Medicine, Okayama University Hospital, 2-5-1 Shikata-cho, Kita-ku, Okayama 700-8558, Japan.
E-mail: htazawa@md.okayama-u.ac.jp

 © 2020 The Author(s).
This is an open access article under the CC BY license (http://creativecommons.org/licenses/by/4.0/).

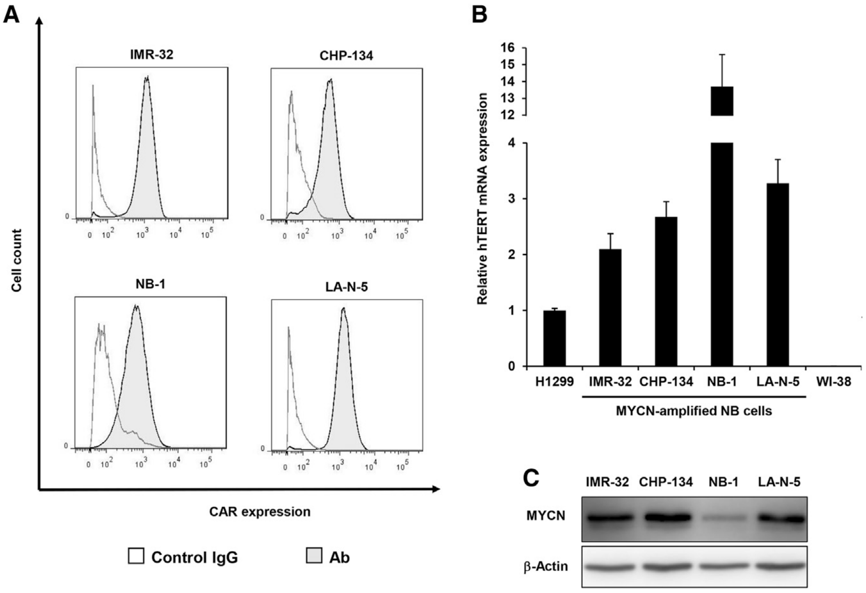

Figure 1. Expression of CAR Protein and Human Telomerase Reverse Transcriptase (hTERT) mRNA in Human NB Cells Exhibiting MYCN Amplification

(A) Expression of CAR protein in human NB cells was analyzed using flow cytometry. Cells were incubated with mouse anti-CAR monoclonal antibody, followed by detection with an FITC-labeled secondary antibody. Isotype-matched normal mouse IgG was used as a control. (B) Expression of hTERT mRNA was analyzed using qRT-PCR. The expression level of hTERT mRNA was calculated relative to that of hTERT mRNA in H1299 cells, which was set at 1. Data are expressed as mean ± SD (n = 3). (C) Expression of MYCN protein in human NB cells was analyzed using western blotting. β-Actin was assayed as a loading control.

underlying the virus-induced antitumor effect, we assessed the modulation of apoptosis- and autophagy-related markers and MYCN in virus-infected NB cells. Finally, the *in vivo* antitumor effect of the oncolytic adenoviruses was evaluated using a subcutaneous NB xenograft tumor model.

RESULTS

Expression of CAR and hTERT in MYCN-Amplified NB Cells

Adenovirus serotype 5 (Ad5) enters target cells via binding of the viral fiber knob to the coxsackievirus and adenovirus receptor (CAR) protein.[17] To evaluate the therapeutic potential of the hTERT-driven oncolytic adenoviruses, which are generated based on the Ad5 genome, in NB cells, we measured the expression level of cell surface CAR protein in four human MYCN-amplified NB cell lines (IMR-32, CHP-134, NB-1, LA-N-5) using flow cytometry analysis. All of the NB cell lines exhibited CAR expression on the cell surface (Figure 1A). Next, we measured the expression level of hTERT mRNA in MYCN-amplified NB cells using real-time RT-PCR analysis. Compared to human lung cancer H1299 cells, all of the NB cell lines exhibited approximately 2- to 13-fold higher expression of hTERT mRNA (Figure 1B). In contrast, no hTERT mRNA expression was detected in normal human lung fibroblast WI38 cells (Figure 1B). Moreover, we confirmed the expression of MYCN protein in the MYCN-amplified NB cell lines by western blot (Figure 1C). The observed expression of CAR and hTERT suggests that MYCN-amplified NB cells are sensitive to hTERT-driven oncolytic adenoviruses.

In Vitro Cytopathic Effect of hTERT-Driven Oncolytic Adenoviruses against MYCN-Amplified NB Cells

To investigate the therapeutic potential of the hTERT-driven oncolytic adenoviruses against MYCN-amplified NB cells, the viability of NB cells was evaluated on day 3 after virus infection using an sodium 3'-[1-(phenylaminocarbonyl)-3,4-tetrazolium]-bis (4-methoxy-6-nitro) benzene sulfonic acid hydrate (2,3-bis-(2-methoxy-4-nitro-5-sulfophenyl)2*H*-tetrazolium-5-carboxanilide) assay. Both OBP-301 and OBP-702 significantly decreased the viability of all NB cells in a dose-dependent manner (Figure 2A; Figure S1). The viability of IMR-32 and LA-N-5 cells was further decreased on day 5 after virus infection (Figure S2). Compared to OBP-301, OBP-702 exhibited a stronger antitumor effect against all NB cell lines examined. As SK-N-SH cells have been reported as non-MYCN-amplified NB cells with high hTERT expression,[18] we analyzed the expression of hTERT mRNA and virus sensitivity in SK-N-SH cells. Non-MYCN-amplified SK-N-SH cells showed high hTERT expression and were sensitive to OBP-301 and OBP-702 as similar with MYCN-amplified NB cells (Figures S3 and S4A). These results suggest that hTERT-expressing NB cells are sensitive to OBP-301 and OBP-702.

To explore the underlying mechanism of the virus-mediated antitumor effect against MYCN-amplified NB cells, we investigated the expression of apoptosis- and autophagy-related proteins on day 3 after virus infection using western blot analysis. No increase in expression of the apoptosis-related marker cleaved poly(ADP-ribose) polymerase (PARP) protein was observed after infection with OBP-301 or OBP-702 (Figure 2B). In contrast, both OBP-301 and OBP-702 induced an increase in expression of the autophagy-related marker LC3-II protein, which is converted from LC3-I protein during autophagy induction. However, the expression of p62 was not detected in NB cells (data not shown). Expression of adenoviral E1A protein was increased in all NB cells infected with either OBP-301 or OBP-702, whereas p53 expression was decreased by OBP-301 and increased by OBP-702. In contrast, non-MYCN-amplified NB cells showed apoptosis and autophagy after virus infection (Figure S4B). These results suggest that the antitumor effect of both OBP-301 and OBP-702 is associated with the induction of autophagy in MYCN-amplified NB cells.

hTERT-Driven Oncolytic Adenoviruses Downregulate MYCN Expression

MYCN plays a crucial role in maintaining the malignant potential of MYCN-amplified NB cells. Therefore, we evaluated

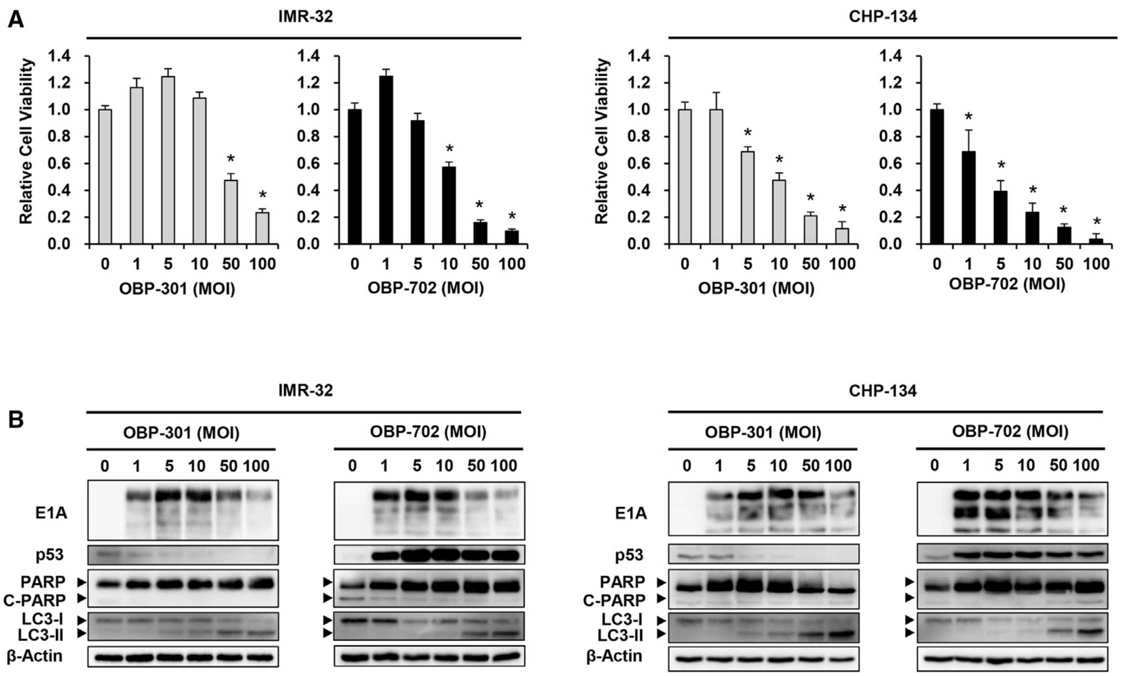

Figure 2. *In Vitro* Cytopathic Effect of OBP-301 and OBP-702 in Association with Autophagy in Human NB Cells

(A) IMR-32 and CHP-134 cells were infected with OBP-301 or OBP-702 at the indicated MOI, and cell viability was evaluated using an XTT assay on day 3 after infection. Cell viability was calculated relative to that of mock-infected cells, which was set at 1.0. Cell viability data are expressed as mean ± SD (n = 5). *$p < 0.05$ (versus an MOI of 0). (B) Expression of viral E1A, p53, PARP, cleaved PARP (C-PARP), and microtubule-associated protein 1 light chain 3 (LC3) protein in IMR-32 and CHP-134 cells infected with OBP-301 or OBP-702 at the indicated MOI for 72 h. β-Actin was assayed as a loading control.

the expression level of MYCN mRNA and protein in MYCN-amplified NB cells infected with OBP-301 or OBP-702. The expression of both MYCN mRNA and protein was downregulated in a dose-dependent manner in OBP-301- and OBP-702-infected NB cells (Figures 3A and 3B). To explore the underlying mechanism of virus-induced MYCN downregulation, we examined the role of E2F1, which is the downstream mediator of viral E1A protein. E2F1 expression was upregulated in a dose-dependent manner in OBP-301- and OBP-702-infected NB cells (Figure 3B). A significant reverse correlation was observed between the expression of E2F1 and MYCN mRNA and protein in OBP-301- and OBP-702-infected NB tumor cells (Figure 3C; Figure S5). These results suggest that E2F1 plays a role in virus-mediated downregulation of MYCN expression.

E2F1 Is Involved in Oncolytic Adenovirus-Induced MYCN Suppression

To explore the relationship between MYCN and E2F1, we used the E2F1-expressing non-replicating adenovirus Ad-E2F1. Ectopic expression of E2F1 induced by Ad-E2F1 downregulated the expression of MYCN mRNA and protein in IMR-32 and CHP-134 cells (Figures 4A–4C). Pretreatment with E2F1 small interfering RNA (siRNA) attenuated the MYCN downregulation in Ad-E2F1-infected NB cells via E2F1 suppression (Figure 4D). In contrast, no suppression of MYCN protein expression was observed in NB cells infected with the E1A deletion control adenovirus dl312 (Figure S6). These results suggest that virus-mediated E2F1 activation downregulates MYCN expression in MYCN-amplified NB cells.

OBP-702-Mediated p53 Activation Is Attenuated during the Late Phase of Virus Infection

We previously demonstrated that OBP-702 induces a more profound antitumor effect than OBP-301 in various types of tumor cells via p53 activation.[11,14] Therefore, we hypothesized that the OBP-702-induced antitumor effect is associated with p53 activation in MYCN-amplified NB cells. We prepared protein lysates from IMR-32 and CHP-134 cells at 0, 24, 48, and 72 h after infection with OBP-301 or OBP-702. Adenoviral E1A expression was increased at 24–48 h, and expression of the downstream mediator E2F1 was increased at 48–72 h after infection with OBP-301 or OBP-702 (Figure 5). In OBP-301-infected NB cells, MYCN expression was decreased at 48 h, consistent with E2F1 upregulation. In contrast, in OBP-702-infected NB cells, MYCN expression was decreased at 24 h, consistent with p53 upregulation, and MYCN suppression was enhanced at 48–72 h, consistent with E2F1 upregulation, even though p53 expression was decreased. These results suggest that E2F1 rather than p53 is mainly involved in OBP-702-mediated MYCN downregulation.

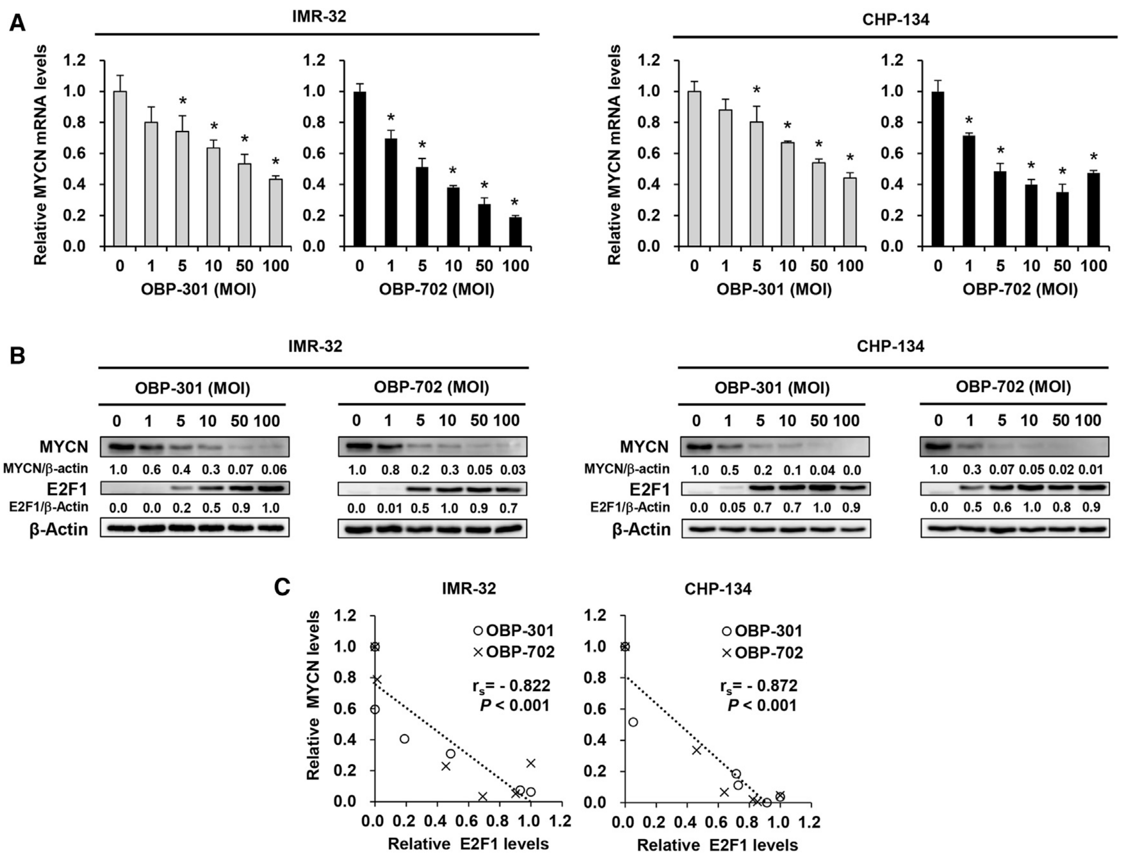

Figure 3. OBP-301 and OBP-702 Induce MYCN Downregulation in Association with E2F1 Upregulation

(A) Expression of MYCN mRNA in IMR-32 and CHP-134 cells infected with OBP-301 or OBP-702 at the indicated MOI for 72 h. The value of MYCN mRNA in mock-infected cells was set at 1, and the relative level of MYCN mRNA in infected cells was plotted as fold induction. Data are expressed as mean ± SD (n = 3). *p < 0.05 (versus an MOI of 0). (B) Expression of MYCN and E2F1 protein in IMR-32 and CHP-134 cells infected with OBP-301 or OBP-702 at the indicated MOI for 72 h. β-Actin was assayed as a loading control. The expression level of MYCN protein was calculated relative to that of mock-infected cells, which was set at 1.0. The expression of E2F1 protein was calculated relative to that of the most E2F1-active cells, which was set at 1.0. (C) Correlation between the expression levels of E2F1 and MYCN protein in IMR-32 and CHP-134 cells infected with OBP-301 or OBP-702.

In Vivo Antitumor Effect of hTERT-Driven Oncolytic Adenoviruses in a Subcutaneous CHP-134 Xenograft Tumor Model

Finally, to assess the *in vivo* antitumor effect of the hTERT-driven oncolytic adenoviruses, we used a subcutaneous CHP-134 xenograft tumor model. We performed two *in vivo* experiments using different protocols of virus treatment. OBP-301, OBP-702, or PBS was injected intratumorally one time a week or three times a week for three cycles. Although three administrations of viruses were not enough to suppress tumor growth (Figure S7), nine administrations of OBP-301 or OBP-702 significantly suppressed tumor growth compared to PBS in the CHP-134 tumor xenograft model (Figures 6A and 6B). However, there was no statistically significant difference in tumor volume between OBP-301 and OBP-702 treatment. Immunohistochemical analyses revealed decreased levels of MYCN expression in both OBP-301- and OBP-702-treated tumors (Figure 6C). Furthermore, compared to control tumors, virus-treated tumors exhibited a significant decrease in the percentage of Ki67-positive proliferating cells (Figure 6D). In contrast, the p53 expression was not detected in CHP-134 tumors (Figure S8). These results suggest that frequent administrations of OBP-301 and OBP-702 have high therapeutic potential for use in eliminating MYCN-amplified NB tumors.

DISCUSSION

Recent comprehensive genomic analyses demonstrated that high-risk NB tumors are characterized by MYCN amplification and/or hTERT rearrangement, which contributes to high hTERT expression and subsequent unfavorable prognosis. In this study, we confirmed that the hTERT-driven oncolytic adenoviruses OBP-301 and OBP-702 have therapeutic antitumor potential against MYCN-amplified NB cells exhibiting high hTERT expression via the induction of autophagy. The virus-mediated antitumor effect is associated with MYCN downregulation, involving virus-induced E2F1 activation. Thus, hTERT-targeted oncolytic virotherapy appears to be a promising antitumor strategy for eliminating MYCN-amplified NB tumor cells via MYCN suppression.

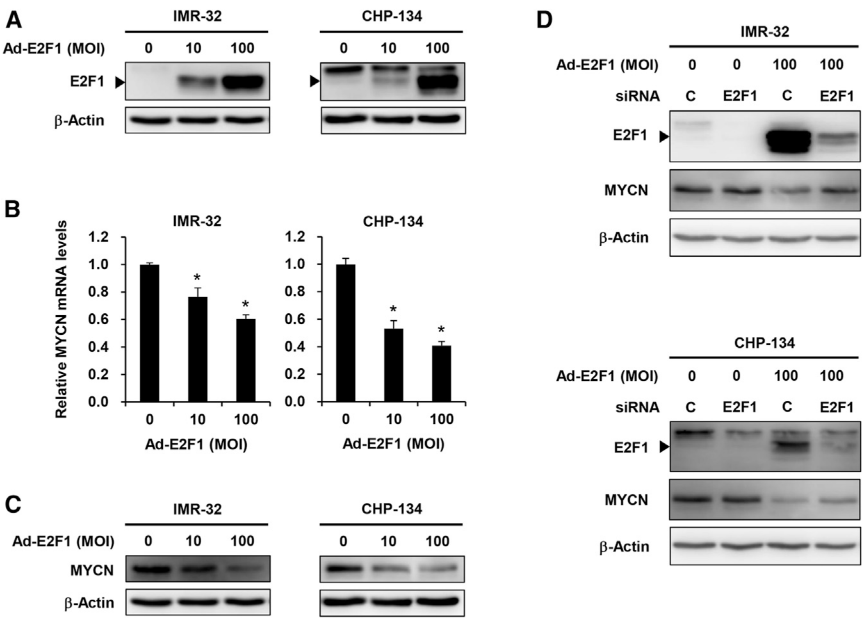

Figure 4. E2F1 Activation Induces MYCN Downregulation

(A) Expression of E2F1 protein in IMR-32 and CHP-134 cells infected with Ad-E2F1 at the indicated MOI for 72 h. (B) Expression of MYCN mRNA in IMR-32 and CHP-134 cells infected with Ad-E2F1 at the indicated MOI for 72 h. The value of MYCN mRNA expression in mock-infected cells was set at 1, and the relative level of MYCN mRNA in infected cells was plotted as fold induction. Data are expressed as mean ± SD (n = 3; *$p < 0.05$ [versus an MOI of 0]). (C) Expression of MYCN protein in IMR-32 and CHP-134 cells infected with Ad-E2F1 at the indicated MOI for 72 h. (D) Expression of MYCN and E2F1 protein in IMR-32 and CHP-134 cells pretreated with control siRNA or E2F1 siRNA (100 nM) before mock infection or Ad-E2F1 infection (100 MOI). β-Actin was assayed as a loading control.

All MYCN-amplified NB cells exhibited high hTERT expression. Although NB-1 cells showed the highest expression of hTERT mRNA, they had the lowest expression of MYCN protein. Recent reports have shown that only NB-1 cells have hTERT promoter mutation among four MYCN-amplified NB cells.[19,20] In addition, non-MYCN-amplified SK-N-SH cells have an hTERT promoter mutation.[20] SK-N-SH cells were sensitive to OBP-301 and OBP-702, similar to MYCN-amplified NB-1 cells. As hTERT promoter mutation is frequently observed in specific types of cancers with high hTERT expression,[21] hTERT-driven oncolytic adenoviruses may be effective to human cancer cells with hTERT promoter mutation.

Autophagy is a catabolic process that induces the lysosomal degradation of intracellular organelles to generate nutrients. In tumor biology, autophagy plays dual roles associated with both cytoprotective and cytotoxic effects. Belounis et al.[22] reported that chemotherapy induces autophagy associated with survival in NB cells, contributing to apoptosis suppression and chemoresistance. In contrast, oncolytic adenoviruses have been shown to induce autophagy associated with cell death in tumor cells via the induction of excessive autophagy.[23] In our study, OBP-301 and OBP-702 exhibited an antitumor effect in association with the autophagy-related death of MYCN-amplified NB cells. We previously reported that OBP-301 induces autophagy-related death of human lung cancer cells.[24] Moreover, the mTOR inhibitor rapamycin improved the antitumor effect of the hTERT-driven oncolytic adenovirus OBP-405 with modified fibers against brain tumor cells via enhanced autophagy.[25] Combining hTERT-driven oncolytic adenoviruses and an autophagy-inducing mTOR inhibitor could be a more effective strategy for inducing the death of MYCN-amplified NB cells.

MYCN plays a critical role in maintaining the malignant potential of NB tumors via the activation of survival, proliferation, metastasis, and angiogenesis processes.[4] Therefore, MYCN inhibition is an attractive therapeutic strategy for eliminating MYCN-amplified NB cells. In this study, the hTERT-driven oncolytic adenoviruses OBP-301 and OBP-702 efficiently suppressed MYCN expression to produce an antitumor effect. Consistent with our data, Li et al.[26] demonstrated that oncolytic adenoviruses with a short-hairpin RNA targeting MYCN exert an antitumor effect against MYCN-amplified NB cells by reducing MYCN expression. Regarding the underlying mechanism of virus-mediated MYCN suppression, we demonstrated that E2F1 upregulation downregulates MYCN expression at both the mRNA and protein levels in OBP-301- and OBP-702-infected NB cells. Some reports have described a possible relationship between MYCN and E2F1. Verena et al.[27] demonstrated that E2F family proteins repress MYCN expression at the transcriptional level in NB cells by binding to the promoter region of the *MYCN* gene. In contrast, Strieder and Lutz[28] reported that the E2F1-induced microRNA-202 suppresses MYCN expression in NB cells at the posttranscriptional level. Although the underlying mechanism of E2F1-mediated MYCN downregulation during oncolytic virotherapy remains unclear, our data suggest that hTERT-driven oncolytic adenoviruses are promising antitumor reagents for eliminating MYCN-amplified NB cells via E2F1-mediated MYCN downregulation.

OBP-702-mediated p53 activation downregulated MYCN expression during the early phase (24 h after infection). However, OBP-702-mediated p53 activation was attenuated during the late phase (48 and 72 h after infection). Moreover, the antitumor effect of OBP-301 and OBP-702 was similar in our subcutaneous xenograft tumor model, suggesting that MYCN-amplified NB cells are relatively insensitive to p53 activation. Although p53 mutations are rare in NB tumors, occurring in less than 2% of cases at diagnosis,[29,30] p53 is often functionally inactivated in MYCN-amplified NB tumors via both direct and indirect mechanisms. With regard to the direct mechanism

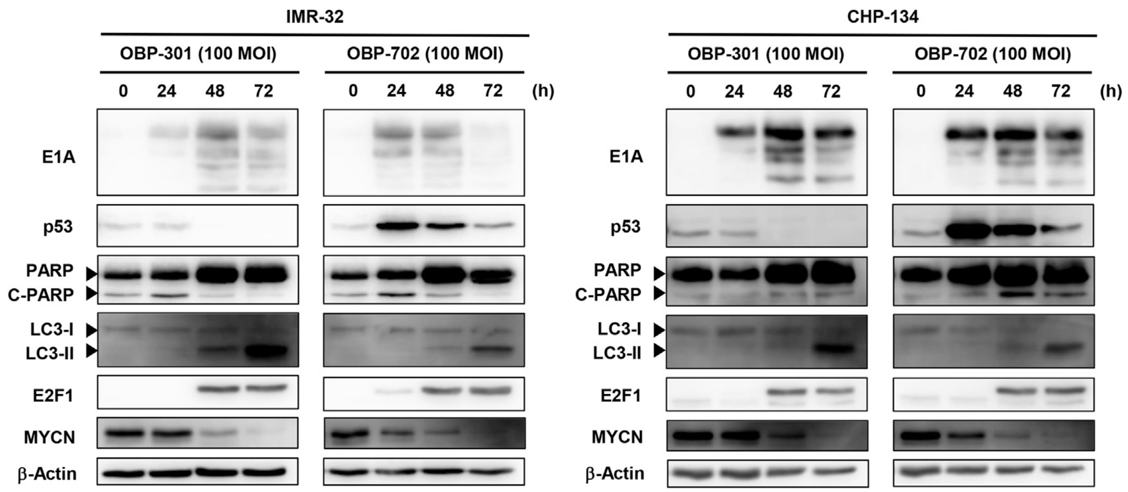

Figure 5. OBP-702-Mediated p53 Activation Is Not Involved in MYCN Downregulation

IMR-32 and CHP-134 cells were infected with OBP-301 or OBP-702 at an MOI of 100, and cells were harvested at the indicated time points. The levels of E1A, p53, PARP, C-PARP, LC3-I, LC3-II, E2F1, and MYCN protein in cell lysates were analyzed by western blotting. β-Actin was assayed as a loading control.

of inactivation, the binding of MYCN to the C-terminal region of p53 results in the inactivation of target genes downstream of p53.[31] With regard to the indirect mechanism in contrast, MYCN-mediated MDM2 activation suppresses p53 expression in MYCN-amplified NB cells via the ubiquitin-proteasome pathway.[32,33] Thus, MYCN-amplified NB cells may be less sensitive to p53-mediated MYCN suppression compared to E2F1-mediated MYCN suppression.

Personalized medicine approaches based on genetic alterations have recently emerged for the treatment of various high-risk pediatric cancers, including NB.[34] Although MYCN activation is the most promising therapeutic target in the treatment of high-risk NB patients, efforts to develop drugs that directly target MYCN have not been successful.[6] Therefore, the development of inhibitors that indirectly target MYCN-related signaling pathways has been challenging.[35] In this study, we confirmed the *in vitro* and *in vivo* therapeutic potential of telomerase-targeted oncolytic adenoviruses against MYCN-amplified NB cells. MYCN suppression is a crucial factor in the underlying mechanism of the virus-mediated antitumor effect. Recent comprehensive genomic analyses demonstrated that hTERT is commonly activated in high-risk NB patients exhibiting MYCN amplification[36] or hTERT rearrangement.[7,8] Therefore, our novel hTERT-driven oncolytic virotherapy could be a particularly useful antitumor strategy for eliminating high-risk NB cells exhibiting MYCN amplification or hTERT rearrangement.

NB tumors most frequently originate from the adrenal gland and retroperitoneal lesions within the body. We can target primary NB tumors by ultrasound imaging or intraoperative injection of oncolytic adenoviruses. To inject these viruses to residual tumors that are unresectable by surgery may be an effective option for the local control of primary NB tumors. However, MYCN-amplified NB cells frequently develop metastatic tumors at the distant organs, which are inaccessible for intratumor injections of oncolytic adenoviruses. Therefore, we need to develop a therapeutic strategy to target inaccessible metastatic NB tumors. Recent evidence has suggested that oncolytic virotherapy induces an antitumor immune response via induction of immunogenic cell death.[37–39] Oncolytic adenoviruses have been shown to induce immunogenic cell death via the release of damage-associated molecular patterns.[40] We recently revealed that intratumoral injection of hTERT-driven oncolytic adenoviruses at the primary site enhances the therapeutic potential of immune checkpoint inhibitors in metastatic colorectal tumors at the liver.[41] Therefore, additional *in vivo* experiments would be warranted to investigate the therapeutic potential of combination therapy with hTERT-driven oncolytic adenoviruses and immunotherapy against metastatic NB tumors.

In conclusion, we demonstrated that use of the telomerase-targeted oncolytic adenoviruses OBP-301 and OBP-702 represents a promising therapeutic approach for the treatment of MYCN-amplified NB tumors via MYCN suppression. To investigate the safety and feasibility of telomerase-targeted oncolytic virotherapy, further clinical study is warranted in high-risk NB patients exhibiting MYCN and hTERT overexpression.

MATERIALS AND METHODS

Cell Lines

The human NB cell lines IMR-32 and NB-1 were obtained from the Japanese Cancer Research Resources Bank (Osaka, Japan). The human NB cell lines CHP-134 and LA-N-5 were purchased from the RIKEN BioResource Center (Tsukuba, Japan). These four NB cell lines were previously reported as MYCN-amplified NB cells.[42,43] The human NB cell line SK-N-SH was obtained from the RIKEN BioResource Center. SK-N-SH cells were used as non-MYCN-amplified NB cells.[18] All NB cell lines have a wild-type *p53* gene, as shown by a

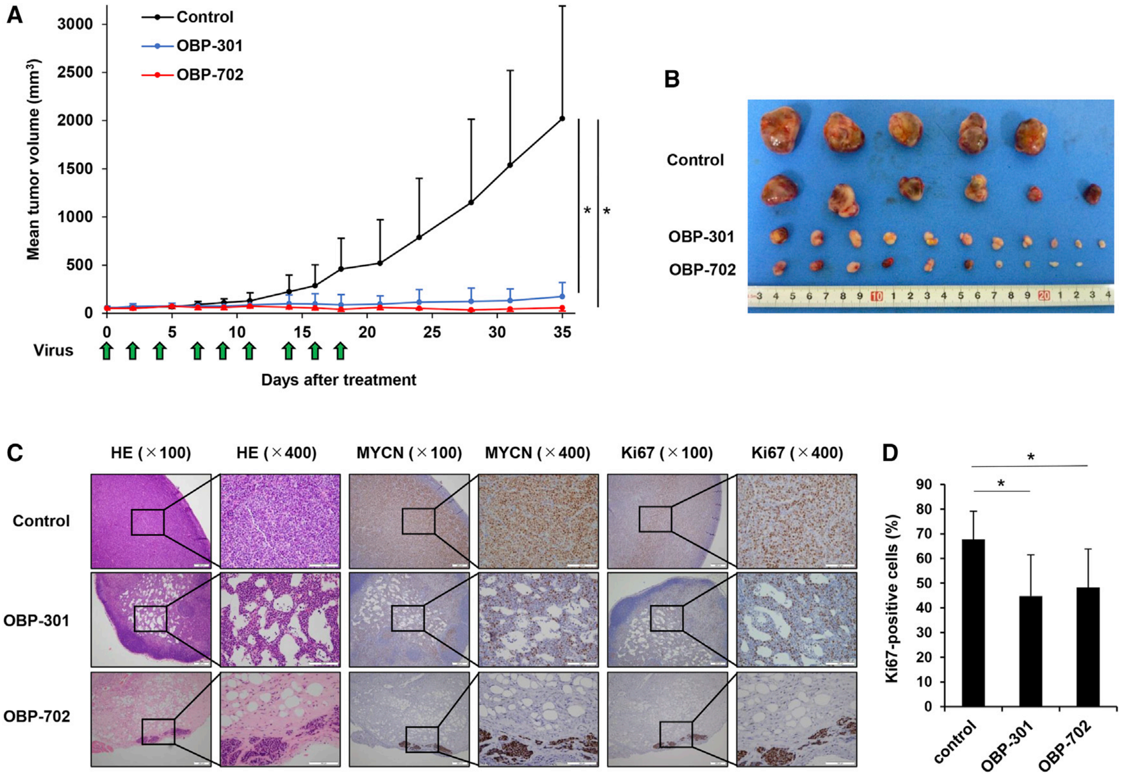

Figure 6. OBP-301 and OBP-702 Inhibit Tumor Growth in a Subcutaneous CHP-134 Xenograft Tumor Model

CHP-134 cells (1×10^7 cells) were inoculated into the flank of 6-week-old female BALB/c *nu/nu* mice. OBP-301 (10^8 PFU), OBP-702 (10^8 PFU), or PBS (mock) was intratumorally injected three times a week for three cycles. (A) Tumor growth is expressed as mean tumor volume ± SD (n = 10 or 11 in each group; *$p < 0.05$). (B) Macroscopic appearance of all isolated tumors in each group. (C) Histologic analysis of CHP-134 tumors. Tumor tissues were obtained at 7 weeks after tumor inoculation. Paraffin-embedded sections of CHP-134 tumors were stained with hematoxylin and eosin (H&E) solution, anti-MYCN antibody, or anti-Ki67 antibody. Left, middle, and right images show H&E, MYCN, and Ki67 staining, respectively. Left-side images in each figure are low magnification, and right-sides images are high magnification of the area outlined by a black square. Left scale bars, 200 μm. Right scale bars, 100 μm. (D) Percentage of Ki67-positive cells in tumor tissue was calculated using ImageJ software. Data are expressed as mean ± SD (n = 5 in each group; *$p < 0.05$).

recent report.[44] The human non-small-cell lung cancer cell line H1299 was obtained from the American Type Culture Collection (Manassas, VA, USA). The human normal fibroblast cell line WI-38 was purchased from the Health Science Research Resources Bank (Osaka, Japan). Cells were cultured for no longer than 5 months following resuscitation. Authentication was not performed by the authors. We confirmed that there was no mycoplasma contamination in all cell lines using PCR-based Myco Finder analysis (Nissui Pharmaceutical) according to the manufacturer's protocol. IMR-32 cells were maintained in Eagle's minimal essential medium with non-essential amino acids and 10% fetal bovine serum (FBS). CHP-134, LA-N-5, and H1299 cells were maintained in RPMI 1640 supplemented with 10% FBS. NB-1 cells were cultured in RPMI 1640 (45%) with Eagle's minimal essential medium (45%) and 10% FBS. SK-N-SH cells were maintained in minimal essential medium alpha with 10% FBS. WI-38 cells were maintained in Eagle's minimal essential medium with 10% FBS. All media were supplemented with 100 U/mL penicillin and 100 mg/mL streptomycin. Cells were routinely maintained at 37°C in a humidified atmosphere with 5% CO_2.

Recombinant Adenoviruses

The telomerase-specific replication-competent adenovirus OBP-301 (Telomelysin), in which the promoter element of the *hTERT* gene derives expression of the *E1A* and *E1B* genes, was previously constructed and characterized.[10,12] OBP-702 was constructed by modifying OBP-301 to express the exogenous *p53* gene by inserting a human wild-type *p53* gene expression cassette driven by the Egr-1 promoter into the E3 region of OBP-301.[11] Replication-deficient adenovirus vectors expressing E2F1 (Ad-E2F1) were used to induce E2F1 expression in the infected cells, as previously reported.[45] An E1A-deletion adenovirus vector (dl312) was used as a control vector. Recombinant adenoviruses were purified using cesium chloride step gradients, and virus titers were determined using a plaque-forming assay with 293 cells. Viruses were stored at −80°C.

Flow Cytometry Analysis

Cells (5×10^4) were labeled with mouse anti-CAR monoclonal antibody (mAb) (RmcB; Upstate Biotechnology, Lake Placid, NY, USA) for 30 min at 4°C and then incubated with secondary fluorescein

isothiocyanate (FITC)-conjugated rabbit anti-mouse immunoglobulin G (IgG) (Zymed Laboratories, South San Francisco, CA, USA). Labeled cells were analyzed using flow cytometry (fluorescence-activated cell sorting [FACS] array; Becton Dickinson, San Jose, CA, USA).

Cell Viability Assay

Cells were seeded in 96-well plates at a density of 5×10^3 cells/well or 2×10^4 cells/well 24 h before virus infection. Cells were infected with OBP-301 or OBP-702 at a multiplicity of infection (MOI) of 0, 1, 10, 50, or 100 plaque-forming units (PFU)/cell. Cell viability was determined on day 3 after virus infection using a Cell Proliferation Kit II (Roche, Indianapolis, IN, USA) according to the manufacturer's protocol.

Western Blot Analysis

IMR-32 and CHP-134 cells were seeded in a 100-mm dish at a density of 1×10^6 and 2×10^6 cells/dish, respectively, 24 h before virus infection. Cells were infected with OBP-301, OBP-702, or Ad-E2F1 at the indicated MOI for 72 h. Whole-cell lysates were prepared in lysis buffer (50 mM Tris-HCl [pH 7.4], 150 mM NaCl, and 1% Triton X-100) containing a protease inhibitor cocktail (Complete Mini; Roche). Proteins were electrophoresed on 6%–15% SDS polyacrylamide gels and transferred onto polyvinylidene fluoride membranes (Hybond-P; GE Healthcare, Buckinghamshire, UK). Blots were blocked with Blocking One (Nacalai Tesque, Kyoto, Japan) at room temperature for 30 min. The primary antibodies used were as follows: rabbit anti-MYCN mAb (Cell Signaling Technology, Danvers, MA, USA), mouse anti-Ad5 E1A mAb (BD Pharmingen, Franklin Lakes, NJ, USA), mouse anti-p53 mAb (Santa Cruz Biotechnology, Santa Cruz, CA, USA), rabbit anti-PARP polyclonal antibody (pAb) (Upstate Biotechnology, Temecula, CA, USA), rabbit anti-microtubule-associated protein 1 light chain 3 (LC3) pAb (Medical & Biological Laboratories [MBL], Nagoya, Japan), rabbit anti-E2F1 mAb (Cell Signaling Technology), and mouse anti-β-actin mAb (Sigma-Aldrich). The secondary antibodies used were horseradish peroxidase-conjugated antibodies against rabbit IgG (GE Healthcare) or mouse IgG (GE Healthcare). Immunoreactive bands on the blots were visualized using enhanced chemiluminescence substrate (ECL Plus; GE Healthcare).

Quantitative Real-Time RT-PCR Analysis

To evaluate the expression of hTERT mRNA, cells were seeded in six-well plates at a density of 2×10^5 cells/well, and after 72 h, total RNA was extracted from the cells using a miRNeasy mini kit (QIAGEN, Valencia, CA, USA). To evaluate the expression of MYCN in virus-infected tumor cells, IMR-32 (2×10^5 cells/well) and CHP-134 cells (4×10^5 cells/well) were seeded in six-well plates 24 h before infection with OBP-301, OBP-702, or Ad-E2F1 at the indicated MOI. Total RNA was extracted from cells on day 3 after infection using a miRNeasy mini kit (QIAGEN). cDNA was synthesized from 10 ng of total RNA using a TaqMan microRNA reverse transcription kit (Applied Biosystems). The expression levels of *hTERT*, *MYCN*, and glyceraldehyde-3-phosphate dehydrogenase (*GAPDH*) mRNA were determined using quantitative real-time PCR with the Applied Biosystems StepOnePlus real-time PCR system (Applied Biosystems). Relative expression levels of *hTERT* mRNA were calculated using the $2^{-\Delta\Delta Ct}$ method after normalization with reference to the expression of *GAPDH* mRNA.

Infection with E2F1-Expressing Adenovirus and Treatment with E2F1 siRNA

IMR-32 or CHP-134 cells were seeded at a density of 5×10^5 or 1×10^6 cells/well in a 60-mm dish and transfected with E2F1 siRNA (Applied Biosystems) or control siRNA (Applied Biosystems) at a concentration of 100 nM. After 24 h, the cells were infected with Ad-E2F1 at an MOI of 100 for 2 days. Whole-cell lysates were prepared from the infected cells, and the expression levels of E2F1 and MYCN protein were analyzed using western blotting.

In Vivo Subcutaneous CHP-134 Xenograft Tumor Model

Animal experimental protocols were approved by the Ethics Review Committee for Animal Experimentation of Okayama University School of Medicine (no. OKU-2015364). CHP-134 xenograft tumors were produced on the left flank of 6-week-old female BALB/c-*nu*/*nu* mice (CLEA Japan, Tokyo, Japan) by subcutaneous injection of 1×10^7 cells in 50 μL of PBS with 50 μL of Matrigel basement membrane matrix (BD Biosciences, Bedford, MA, USA). When the tumors had grown to a diameter of 4–6 mm, OBP-301 (1×10^8 PFU), OBP-702 (1×10^8 PFU), or PBS was injected into the tumors one time a week or three times a week for 3 cycles. Tumor size was monitored by measuring tumor length and width using calipers (n = 10 or 11 in each group). CHP-134 tumor volume was calculated using the following formula: $(L \times W^2) \times 0.5$, where L represents the length and W represents the width of each tumor.

Histopathologic Analysis

Tumors were fixed in 10% neutralized formalin and embedded in paraffin blocks. Paraffin-embedded sections (4 μm) were prepared for hematoxylin and eosin staining and immunohistochemical examination. Immunostaining with rabbit anti-MYCN mAb (Cell Signaling Technology), rabbit anti-Ki67 mAb (Abcam, Cambridge, MA, USA), or rabbit anti-p53 mAb (Cell Signaling Technology) according to standard techniques was used to detect proliferating tumor cells within tumor tissues. Photomicrographs of immunostained sections were obtained under light microscopy. The number of total cells and Ki67-immunoreactive cells was determined from five randomly selected fields in each tumor using ImageJ software.

Statistical Analysis

Data are expressed as mean ± standard deviation (SD). Significant differences were assessed using the Student's t test. One-way ANOVA followed by a Games-Howell multiple-group comparison test was used to compare differences between groups in animal experiments. Correlations between expression levels of E2F1 and MYCN protein were analyzed using Spearman's rank correlation coefficient. Statistical significance was defined as a p value of less than 0.05.

SUPPLEMENTAL INFORMATION

Supplemental Information can be found online at https://doi.org/10.1016/j.omto.2020.05.015.

AUTHOR CONTRIBUTIONS

Conception and design: H.T., S.K., and T.F. Development of methodology: T.T., H.T., and T.I. Acquisition of data (e.g., provided animals, provided facilities): T.T., T.I., H.N., M.T., and T.O. Analysis and interpretation of data (e.g., statistical analysis, biostatistics, computational analysis): T.T., H.T., T.I., H.N., M.T., and T.O. Writing, review, and/or revision of the manuscript: T.T., H.T., and T.F. Administrative, technical, or material support: Y.U. Study supervision: H.T., S.K., T.N., and T.F.

CONFLICTS OF INTEREST

Y.U. is President & CEO of Oncolys BioPharma, the manufacturer of OBP-301 (Telomelysin). H.T. and T.F. are consultants for Oncolys BioPharma. The remaining authors declare no competing interests.

ACKNOWLEDGMENTS

We thank Tomoko Sueishi and Tae Yamanishi for excellent technical support. This study was supported in part by grants from the Japan Agency for Medical Research and Development (to T.F., no. 17ck0106285h001) and the Ministry of Education, Culture, Sports, Science, and Technology, Japan (to T.F., nos. 25293283 and 16H05416; to H.T., no. 16K10596; and to T.T., no. 19K18054).

REFERENCES

1. Maris, J.M., Hogarty, M.D., Bagatell, R., and Cohn, S.L. (2007). Neuroblastoma. Lancet *369*, 2106–2120.
2. Shimada, H., Ambros, I.M., Dehner, L.P., Hata, J., Joshi, V.V., and Roald, B. (1999). Terminology and morphologic criteria of neuroblastic tumors: recommendations by the International Neuroblastoma Pathology Committee. Cancer *86*, 349–363.
3. Cheung, N.K., and Dyer, M.A. (2013). Neuroblastoma: developmental biology, cancer genomics and immunotherapy. Nat. Rev. Cancer *13*, 397–411.
4. Huang, M., and Weiss, W.A. (2013). Neuroblastoma and MYCN. Cold Spring Harb. Perspect. Med. *3*, a014415.
5. Beltran, H. (2014). The N-myc oncogene: maximizing its targets, regulation, and therapeutic potential. Mol. Cancer Res. *12*, 815–822.
6. Sala, A. (2015). Editorial: targeting MYCN in pediatric cancers. Front. Oncol. *4*, 330.
7. Peifer, M., Hertwig, F., Roels, F., Dreidax, D., Gartlgruber, M., Menon, R., Krämer, A., Roncaioli, J.L., Sand, F., Heuckmann, J.M., et al. (2015). Telomerase activation by genomic rearrangements in high-risk neuroblastoma. Nature *526*, 700–704.
8. Valentijn, L.J., Koster, J., Zwijnenburg, D.A., Hasselt, N.E., van Sluis, P., Volckmann, R., van Noesel, M.M., George, R.E., Tytgat, G.A., Molenaar, J.J., and Versteeg, R. (2015). TERT rearrangements are frequent in neuroblastoma and identify aggressive tumors. Nat. Genet. *47*, 1411–1414.
9. Lawler, S.E., Speranza, M.C., Cho, C.F., and Chiocca, E.A. (2017). Oncolytic viruses in cancer treatment: a review. JAMA Oncol. *3*, 841–849.
10. Kawashima, T., Kagawa, S., Kobayashi, N., Shirakiya, Y., Umeoka, T., Teraishi, F., Taki, M., Kyo, S., Tanaka, N., and Fujiwara, T. (2004). Telomerase-specific replication-selective virotherapy for human cancer. Clin. Cancer Res. *10*, 285–292.
11. Yamasaki, Y., Tazawa, H., Hashimoto, Y., Kojima, T., Kuroda, S., Yano, S., Yoshida, R., Uno, F., Mizuguchi, H., Ohtsuru, A., et al. (2012). A novel apoptotic mechanism of genetically engineered adenovirus-mediated tumour-specific p53 overexpression through E1A-dependent p21 and MDM2 suppression. Eur. J. Cancer *48*, 2282–2291.
12. Hashimoto, Y., Watanabe, Y., Shirakiya, Y., Uno, F., Kagawa, S., Kawamura, H., Nagai, K., Tanaka, N., Kumon, H., Urata, Y., and Fujiwara, T. (2008). Establishment of biological and pharmacokinetic assays of telomerase-specific replication-selective adenovirus. Cancer Sci. *99*, 385–390.
13. Sasaki, T., Tazawa, H., Hasei, J., Kunisada, T., Yoshida, A., Hashimoto, Y., Yano, S., Yoshida, R., Uno, F., Kagawa, S., et al. (2011). Preclinical evaluation of telomerase-specific oncolytic virotherapy for human bone and soft tissue sarcomas. Clin. Cancer Res. *17*, 1828–1838.
14. Hasei, J., Sasaki, T., Tazawa, H., Osaki, S., Yamakawa, Y., Kunisada, T., Yoshida, A., Hashimoto, Y., Onishi, T., Uno, F., et al. (2013). Dual programmed cell death pathways induced by p53 transactivation overcome resistance to oncolytic adenovirus in human osteosarcoma cells. Mol. Cancer Ther. *12*, 314–325.
15. Nemunaitis, J., Tong, A.W., Nemunaitis, M., Senzer, N., Phadke, A.P., Bedell, C., Adams, N., Zhang, Y.A., Maples, P.B., Chen, S., et al. (2010). A phase I study of telomerase-specific replication competent oncolytic adenovirus (Telomelysin) for various solid tumors. Mol. Ther. *18*, 429–434.
16. Kuroda, S., Fujiwara, T., Shirakawa, Y., Yamasaki, Y., Yano, S., Uno, F., Tazawa, H., Hashimoto, Y., Watanabe, Y., Noma, K., et al. (2010). Telomerase-dependent oncolytic adenovirus sensitizes human cancer cells to ionizing radiation via inhibition of DNA repair machinery. Cancer Res. *70*, 9339–9348.
17. Bergelson, J.M., Cunningham, J.A., Droguett, G., Kurt-Jones, E.A., Krithivas, A., Hong, J.S., Horwitz, M.S., Crowell, R.L., and Finberg, R.W. (1997). Isolation of a common receptor for coxsackie B viruses and adenoviruses 2 and 5. Science *275*, 1320–1323.
18. O'Brien, R., Tran, S.L., Maritz, M.F., Liu, B., Kong, C.F., Purgato, S., Yang, C., Murray, J., Russell, A.J., Flemming, C.L., et al. (2016). MYC-driven neuroblastomas are addicted to a telomerase-independent function of dyskerin. Cancer Res. *76*, 3604–3617.
19. Ghandi, M., Huang, F.W., Jané-Valbuena, J., Kryukov, G.V., Lo, C.C., McDonald, E.R., 3rd, Barretina, J., Gelfand, E.T., Bielski, C.M., Li, H., et al. (2019). Next-generation characterization of the Cancer Cell Line Encyclopedia. Nature *569*, 503–508.
20. Lindner, S., Bachmann, H.S., Odersky, A., Schaefers, S., Klein-Hitpass, L., Hero, B., Fischer, M., Eggert, A., Schramm, A., and Schulte, J.H. (2015). Absence of telomerase reverse transcriptase promoter mutations in neuroblastoma. Biomed. Rep. *3*, 443–446.
21. Vinagre, J., Almeida, A., Pópulo, H., Batista, R., Lyra, J., Pinto, V., Coelho, R., Celestino, R., Prazeres, H., Lima, L., et al. (2013). Frequency of TERT promoter mutations in human cancers. Nat. Commun. *4*, 2185.
22. Belounis, A., Nyalendo, C., Le Gall, R., Imbriglio, T.V., Mahma, M., Teira, P., Beaunoyer, M., Cournoyer, S., Haddad, E., Vassal, G., and Sartelet, H. (2016). Autophagy is associated with chemoresistance in neuroblastoma. BMC Cancer *16*, 891.
23. Tazawa, H., Kagawa, S., and Fujiwara, T. (2013). Oncolytic adenovirus-induced autophagy: tumor-suppressive effect and molecular basis. Acta Med. Okayama *67*, 333–342.
24. Tazawa, H., Yano, S., Yoshida, R., Yamasaki, Y., Sasaki, T., Hashimoto, Y., Kuroda, S., Ouchi, M., Onishi, T., Uno, F., et al. (2012). Genetically engineered oncolytic adenovirus induces autophagic cell death through an E2F1-microRNA-7-epidermal growth factor receptor axis. Int. J. Cancer *131*, 2939–2950.
25. Yokoyama, T., Iwado, E., Kondo, Y., Aoki, H., Hayashi, Y., Georgescu, M.M., Sawaya, R., Hess, K.R., Mills, G.B., Kawamura, H., et al. (2008). Autophagy-inducing agents augment the antitumor effect of telerase-selve oncolytic adenovirus OBP-405 on glioblastoma cells. Gene Ther. *15*, 1233–1239.
26. Li, Y., Zhang, B., Zhang, H., Zhu, X., Feng, D., Zhang, D., Zhuo, B., Li, L., and Zheng, J. (2013). Oncolytic adenovirus armed with shRNA targeting MYCN gene inhibits neuroblastoma cell proliferation and in vivo xenograft tumor growth. J. Cancer Res. Clin. Oncol. *139*, 933–941.
27. Strieder, V., and Lutz, W. (2003). E2F proteins regulate *MYCN* expression in neuroblastomas. J. Biol. Chem. *278*, 2983–2989.
28. Li, Y.G., He, J.H., Yu, L., Hang, Z.P., Li, W., Shun, W.H., and Huang, G.X. (2014). MicroRNA-202 suppresses MYCN expression under the control of E2F1 in the neuroblastoma cell line LAN-5. Mol. Med. Rep. *9*, 541–546.

29. Vogan, K., Bernstein, M., Leclerc, J.M., Brisson, L., Brossard, J., Brodeur, G.M., Pelletier, J., and Gros, P. (1993). Absence of p53 gene mutations in primary neuroblastomas. Cancer Res. *53*, 5269–5273.

30. Hosoi, G., Hara, J., Okamura, T., Osugi, Y., Ishihara, S., Fukuzawa, M., Okada, A., Okada, S., and Tawa, A. (1994). Low frequency of the p53 gene mutations in neuroblastoma. Cancer *73*, 3087–3093.

31. Agarwal, S., Milazzo, G., Rajapakshe, K., Bernardi, R., Chen, Z., Barbieri, E., Koster, J., Perini, G., Coarfa, C., and Shohet, J.M. (2018). MYCN acts as a direct co-regulator of p53 in MYCN amplified neuroblastoma. Oncotarget *9*, 20323–20338.

32. Carr-Wilkinson, J., O'Toole, K., Wood, K.M., Challen, C.C., Baker, A.G., Board, J.R., Evans, L., Cole, M., Cheung, N.K., Boos, J., et al. (2010). High frequency of p53/MDM2/p14^{ARF} pathway abnormalities in relapsed neuroblastoma. Clin. Cancer Res. *16*, 1108–1118.

33. Slack, A., Chen, Z., Tonelli, R., Pule, M., Hunt, L., Pession, A., and Shohet, J.M. (2005). The p53 regulatory gene *MDM2* is a direct transcriptional target of MYCN in neuroblastoma. Proc. Natl. Acad. Sci. USA *102*, 731–736.

34. Worst, B.C., van Tilburg, C.M., Balasubramanian, G.P., Fiesel, P., Witt, R., Freitag, A., Boudalil, M., Previti, C., Wolf, S., Schmidt, S., et al. (2016). Next-generation personalised medicine for high-risk paediatric cancer patients—the INFORM pilot study. Eur. J. Cancer *65*, 91–101.

35. Greengard, E.G. (2018). Molecularly targeted therapy for neuroblastoma. Children (Basel) *5*, 142.

36. Hiyama, E., Hiyama, K., Yokoyama, T., Matsuura, Y., Piatyszek, M.A., and Shay, J.W. (1995). Correlating telomerase activity levels with human neuroblastoma outcomes. Nat. Med. *1*, 249–255.

37. Jiang, H., and Fueyo, J. (2014). Healing after death: antitumor immunity induced by oncolytic adenoviral therapy. OncoImmunology *3*, e947872.

38. Bartlett, D.L., Liu, Z., Sathaiah, M., Ravindranathan, R., Guo, Z., He, Y., and Guo, Z.S. (2013). Oncolytic viruses as therapeutic cancer vaccines. Mol. Cancer *12*, 103.

39. Guo, Z.S., Liu, Z., and Bartlett, D.L. (2014). Oncolytic immunotherapy: dying the right way is a key to eliciting potent antitumor immunity. Front. Oncol. *4*, 74.

40. Tazawa, H., Kuroda, S., Hasei, J., Kagawa, S., and Fujiwara, T. (2017). Impact of Autophagy In Oncolytic Adenoviral Therapy For Cancer. Int. J. Mol. Sci. *18*, 1479.

41. Kanaya, N., Kuroda, S., Kakiuchi, Y., Kumon, K., Tsumura, T., Hashimoto, M., Morihiro, T., Kubota, T., Aoyama, K., Kikuchi, S., et al. (2020). Immune modulation by telomerase-specific oncolytic adenovirus synergistically enhances antitumor efficacy with anti-PD1 antibody. Mol. Ther. *28*, 794–804.

42. Amler, L.C., and Schwab, M. (1989). Amplified N-myc in human neuroblastoma cells is often arranged as clustered tandem repeats of differently recombined DNA. Mol. Cell. Biol. *9*, 4903–4913.

43. Nara, K., Kusafuka, T., Yoneda, A., Oue, T., Sangkhathat, S., and Fukuzawa, M. (2007). Silencing of MYCN by RNA interference induces growth inhibition, apoptotic activity and cell differentiation in a neuroblastoma cell line with MYCN amplification. Int. J. Oncol. *30*, 1189–1196.

44. Harenza, J.L., Diamond, M.A., Adams, R.N., Song, M.M., Davidson, H.L., Hart, L.S., Dent, M.H., Fortina, P., Reynolds, C.P., and Maris, J.M. (2017). Transcriptomic profiling of 39 commonly-used neuroblastoma cell lines. Sci. Data *4*, 170033.

45. Itoshima, T., Fujiwara, T., Waku, T., Shao, J., Kataoka, M., Yarbrough, W.G., Liu, T.J., Roth, J.A., Tanaka, N., and Kodama, M. (2000). Induction of apoptosis in human esophageal cancer cells by sequential transfer of the wild-type p53 and E2F-1 genes: involvement of p53 accumulation via ARF-mediated MDM2 down-regulation. Clin. Cancer Res. *6*, 2851–2859.

OMTO, Volume *18*

Supplemental Information

Elimination of MYCN-Amplified Neuroblastoma Cells by Telomerase-Targeted Oncolytic Virus via MYCN Suppression

Terutaka Tanimoto, Hiroshi Tazawa, Takeshi Ieda, Hiroshi Nouso, Morimichi Tani, Takanori Oyama, Yasuo Urata, Shunsuke Kagawa, Takuo Noda, and Toshiyoshi Fujiwara

SUPPLEMENTARY MATERIAL

Elimination of MYCN-amplified neuroblastoma cells by telomerase-targeted oncolytic virus via MYCN suppression

Terutaka Tanimoto[1,2], Hiroshi Tazawa[1,3], Takeshi Ieda[1], Hiroshi Nouso[2], Morimichi Tani[2], Takanori Oyama[2], Yasuo Urata[5], Shunsuke Kagawa[1,3], Takuo Noda[2], and Toshiyoshi Fujiwara[1]

Departments of [1]Gastroenterological Surgery and [2]Pediatric Surgery, Okayama University Graduate School of Medicine, Dentistry and Pharmaceutical Sciences, Okayama 700-8558, Japan. [3]Center for Innovative Clinical Medicine and [4]Minimally Invasive Therapy Center, Okayama University Hospital, Okayama 700-8558, Japan.
[5]Oncolys BioPharma, Inc., Tokyo, 106-0032, Japan.

Figure S1
In vitro cytopathic effect of OBP-301 and OBP-702 against NB-1 and LA-N-5 cells.

Figure S2
In vitro cytopathic effect of OBP-301 and OBP-702 against IMR-32 and LA-N-5 cells.

Figure S3
Expression of hTERT mRNA in human NB cells with or without MYCN amplification.

Figure S4
In vitro cytopathic effect of OBP-301 and OBP-702 against non-MYCN-amplified SK-N-SH cells.

Figure S5
Inverse correlation between E2F1 protein and MYCN mRNA expression.

Figure S6
MYCN is not downregulated in IMR-32 and CHP-134 cells infected with dl312.

Figure S7
No antitumor effect of OBP-301 and OBP-702 in a subcutaneous CHP-134 xenograft tumor model.

Figure S8
Histologic analysis of CHP-134 tumors.

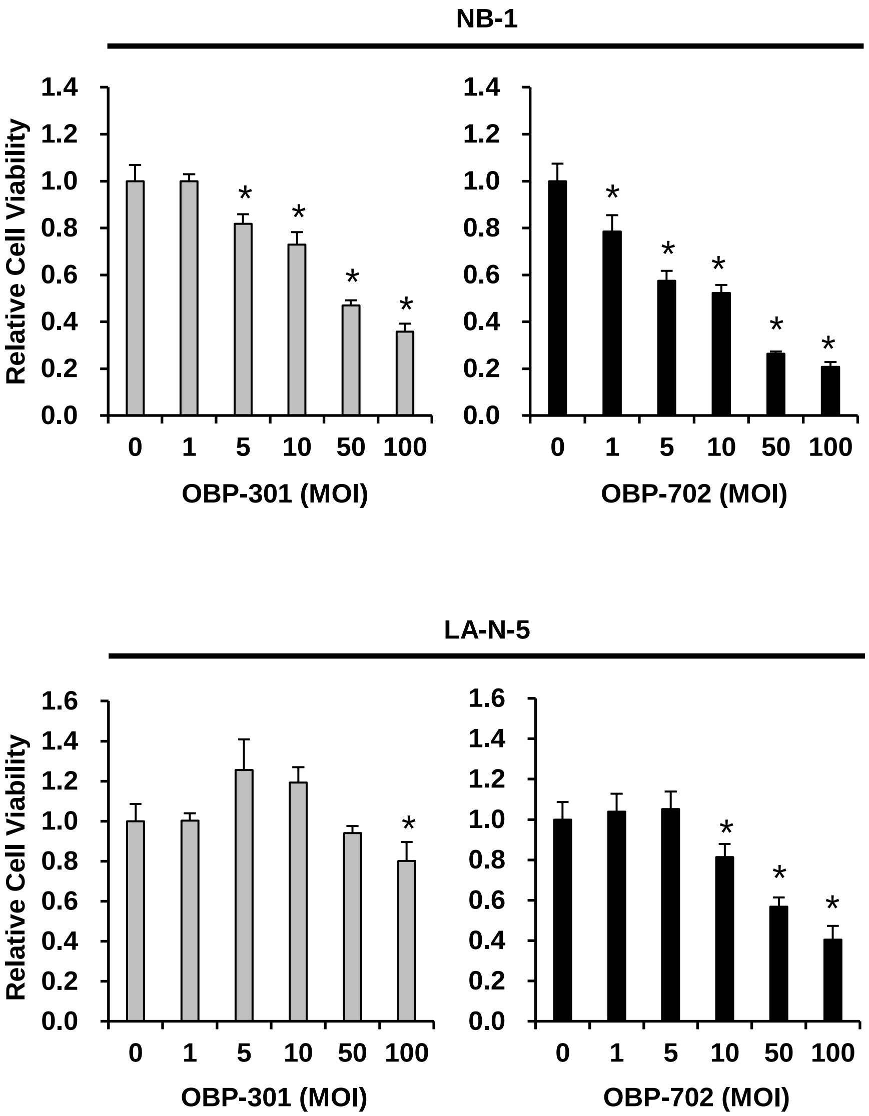

Figure S1

In vitro cytopathic effect of OBP-301 and OBP-702 against NB-1 and LA-N-5 cells.

NB-1 and LA-N-5 cells were infected with OBP-301 or OBP-702 at the indicated MOI, and cell viability was evaluated on day 3 after infection using an XTT assay . Cell viability was calculated relative to that of mock-infected cells, which was set at 1.0. Cell viability data are expressed as mean ± SD (n = 5). *: $P < 0.05$ (vs 0 MOI).

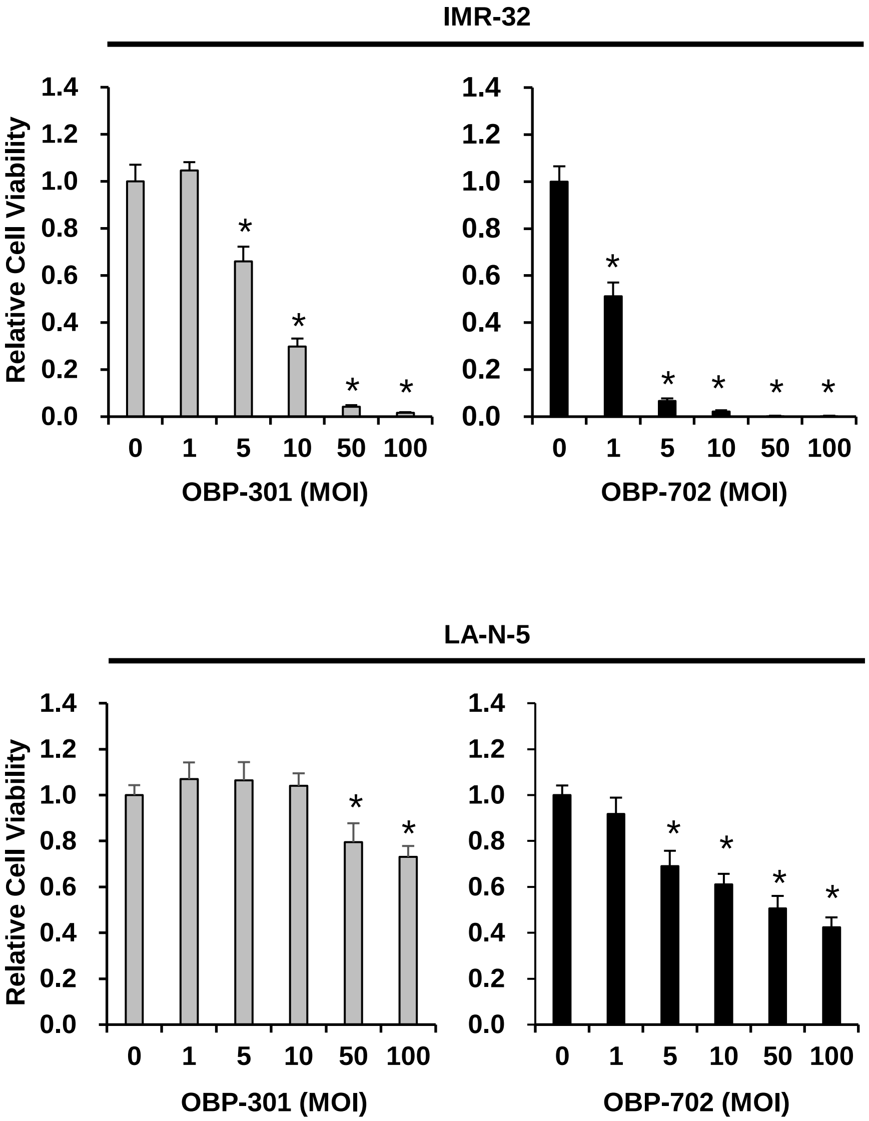

Figure S2

***In vitro* cytopathic effect of OBP-301 and OBP-702 against IMR-32 and LA-N-5 cells.**

IMR-32 and LA-N-5 cells were infected with OBP-301 or OBP-702 at the indicated MOI, and cell viability was evaluated on day 5 after infection using an XTT assay . Cell viability was calculated relative to that of mock-infected cells, which was set at 1.0. Cell viability data are expressed as mean ± SD (n = 5). *: $P < 0.05$ (vs 0 MOI).

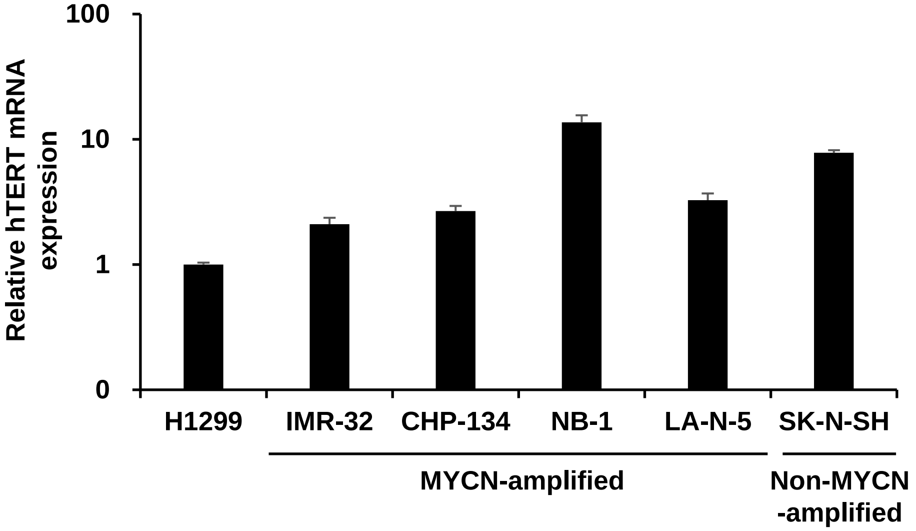

Figure S3

Expression of hTERT mRNA in human NB cells with or without MYCN amplification.

Expression of hTERT mRNA was analyzed using qRT-PCR. The expression level of hTERT mRNA was calculated relative to that of hTERT mRNA in H1299 cells, which was set at 1. Data are expressed as mean ± SD (n = 3).

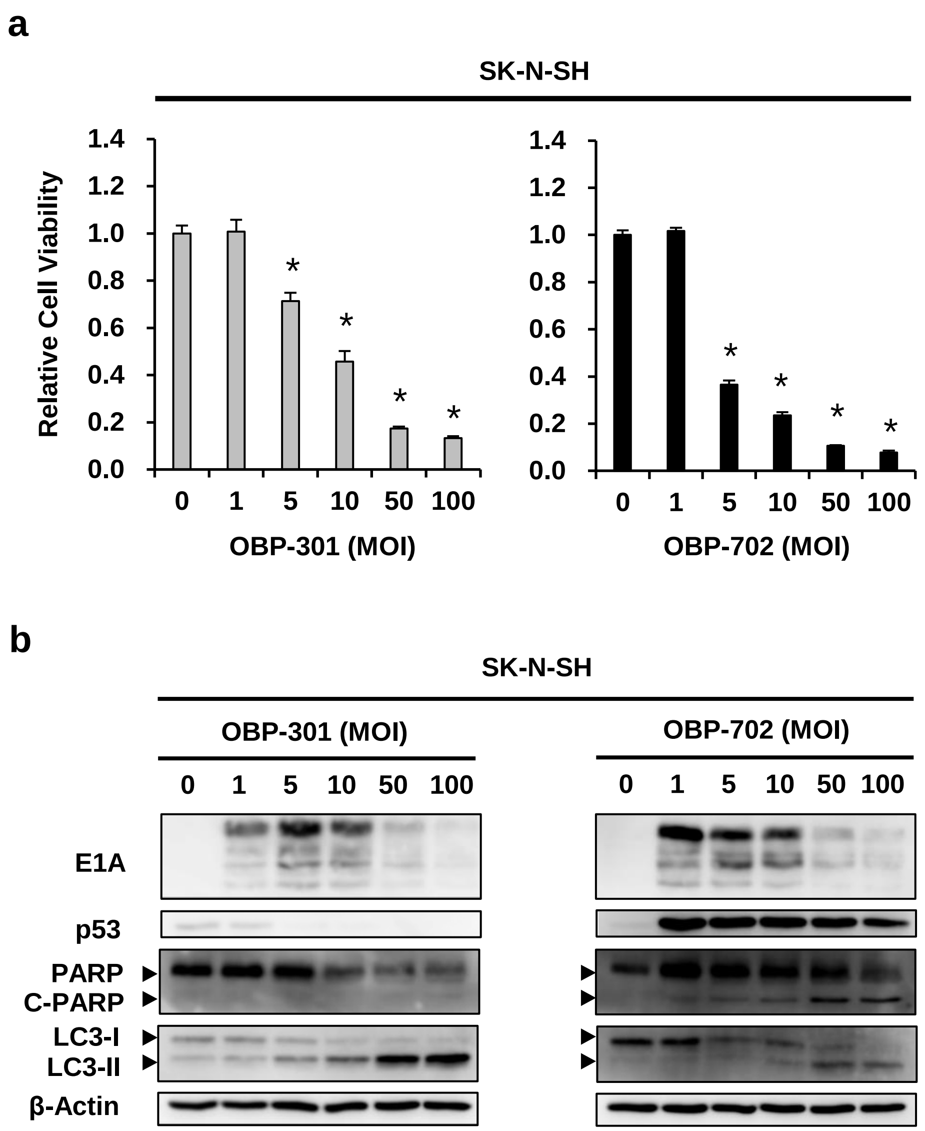

Figure S4

In vitro cytopathic effect of OBP-301 and OBP-702 in non-MYCN-amplified SK-N-SH cells.

(a) SK-N-SH cells were infected with OBP-301 or OBP-702 at the indicated MOI, and cell viability was evaluated on day 3 after infection using an XTT assay. Cell viability was calculated relative to that of mock-infected cells, which was set at 1.0. Cell viability data are expressed as mean ± SD (n = 5). *: $P < 0.05$ (vs 0 MOI). (b) Expression of viral E1A, p53, PARP, cleaved PARP (C-PARP), and LC3 protein in SK-N-SH cells infected with OBP-301 or OBP-702 at the indicated MOI for 72 h. β-Actin was assayed as a loading control.

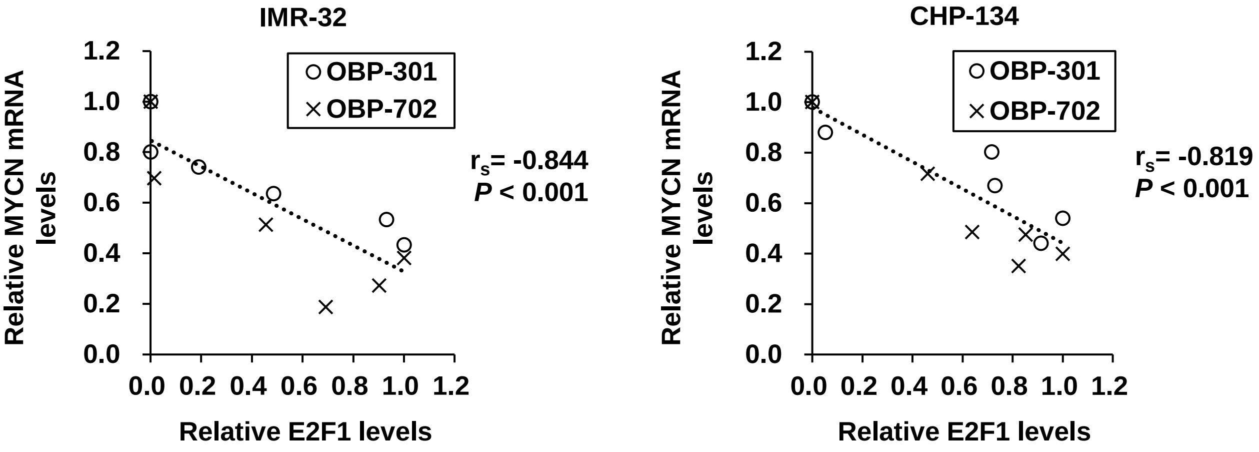

Figure S5

Inverse correlation between E2F1 protein and MYCN mRNA expression.

There was significant correlation between the expression levels of E2F1 protein and MYCN mRNA in IMR-32 and CHP-134 cells. The expression level of E2F1 protein was calculated relative to that of the most E2F1-active cells, which was set at 1.0. The expression level of MYCN mRNA was calculated relative to that of mock-infected cells, which was set at 1.0.

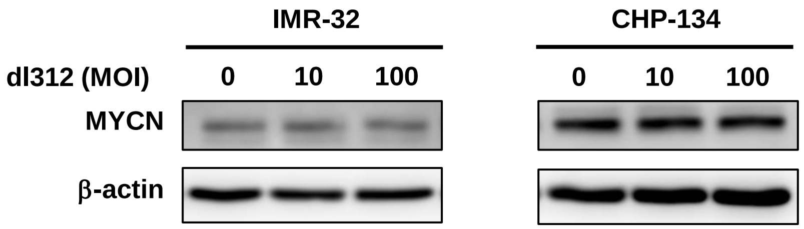

Figure S6

MYCN is not downregulated in IMR-32 and CHP-134 cells infected with dl312.

Expression of MYCN protein in IMR-32 and CHP-134 cells infected with dl312 at the indicated MOI for 72 h. β-Actin was assayed as a loading control.

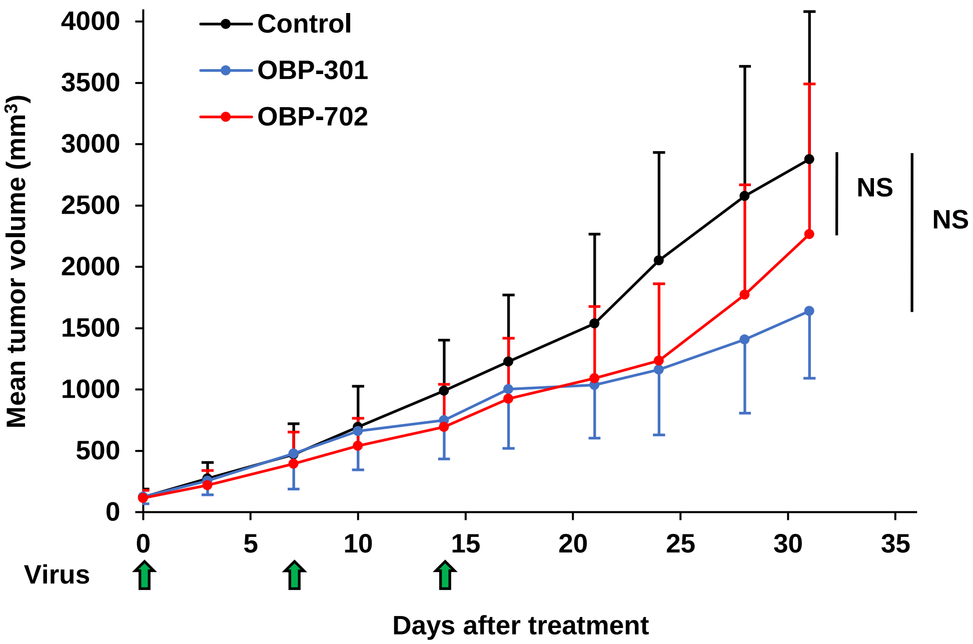

Figure S7

No antitumor effect of OBP-301 and OBP-702 in a subcutaneous CHP-134 xenograft tumor model.

CHP-134 cells (1×10^7 cells) were inoculated into the flank of 6-week-old female BALB/c *nu/nu* mice. OBP-301 (10^8 PFU), OBP-702 (10^8 PFU), or PBS (Mock) was intratumorally injected one time a week for 3 cycles. Tumor growth is expressed as mean tumor volume ± SD (n = 8-10 in each group; NS, not significant).

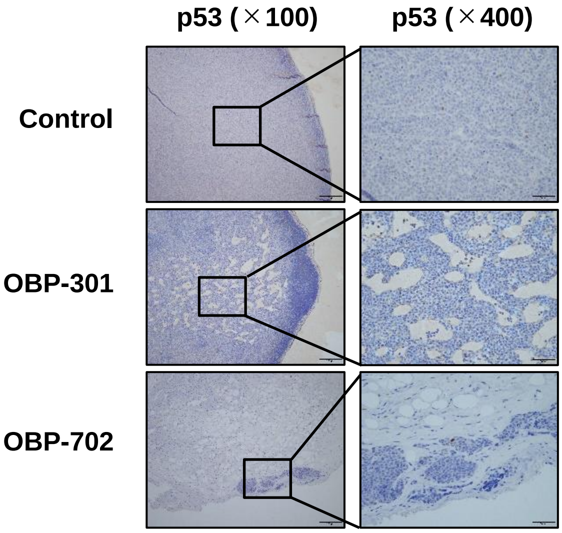

Figure S8

Histologic analysis of CHP-134 tumors.

Paraffin-embedded sections of CHP-134 tumors were stained with anti-p53 antibody. Left-side images in each figure are low magnification, and right-side images are high magnification of the area outlined by a black square. Left scale bars, 200 μm. Right scale bars, 100 μm.
